# Noninvasive Monitoring of Programmed Death-Ligand 2 Expression with Positron Emission Tomography using ^68^Ga-labeled Peptide Antagonist in Preclinical and Exploratory Human Studies

**DOI:** 10.34133/research.0523

**Published:** 2024-11-01

**Authors:** Yajie Zhao, Xiaoqin Yin, Ming Zhou, Wanqian Rao, Xuan Ji, Xiaobo Wang, XiaoXiong Xiao, Shuo Hu

**Affiliations:** ^1^ Department of Nuclear Medicine, Xiangya Hospital, Central South University, Changsha 410008, China.; ^2^ Department of Periodontology, Suzhou Stomatological Hospital, Suzhou, Jiangsu 215026, China.; ^3^ Department of Nuclear Medicine and State Key Laboratory of Holistic Integrative Management of Gastrointestinal Cancers, Xijing Hospital, Fourth Military Medical University, Xi’an 710032, China.; ^4^ Department of Thoracic Surgery, Xiangya Hospital, Central South University, Changsha 410008, China.; ^5^ National Clinical Research Center for Geriatric Disorders (Xiangya), Changsha 410008, China.; ^6^ Key Laboratory of Biological Nanotechnology of National Health Commission, Xiangya Hospital, Central South University, Changsha 410008, China.

## Abstract

While the expression of programmed death ligand-1 (PD-L1) is associated with response to immune therapy, PD-L1-negative patients may still benefit from immune treatment. Programmed death ligand-2 (PD-L2), another crucial immune checkpoint molecule interacting with PD-1, correlates with the efficacy of various tumor immune therapies. This study investigates the expression of PD-L2 in non-small cell lung cancer (NSCLC) patients following anti-PD-1 therapy and its predictive value for clinical survival outcomes. Additionally, we explore the noninvasive, real-time, and dynamic quantitative analysis potential of PD-L2 positron emission tomography (PET) imaging in transplanted tumors. We utilized [^68^Ga]Ga-labeled peptide HN11-1 for PD-L2 PET imaging. The results indicate a higher response rate to anti-PD-1 therapy in patients positive for both PD-L1 and PD-L2, with PD-L2 status independently predicting progression-free survival (PFS) with pembrolizumab treatment. Furthermore, [^68^Ga]Ga-HN11-1 PET imaging demonstrates specificity in assessing PD-L2 status. Overall, we confirm the correlation between high PD-L2 expression and favorable PFS in NSCLC patients post anti-PD-1 therapy and highlight the promising potential of [^68^Ga]Ga-HN11-1 as a specific tracer for PD-L2 in preclinical and initial human trials.

## Introduction

Previous studies have demonstrated that immune checkpoint inhibitors (ICIs) targeting programmed cell death protein 1 (PD-1) can exhibit clinical efficacy in patients with tumors expressing high levels of programmed death ligand-1 (PD-L1) [1–3]. Response rates to anti-PD-1 therapy, whether administered alone or in conjunction with chemotherapy, fluctuate among various solid tumors. Statistics show that these response rates typically range from 28% to 69.8% [1–4]. ICIs have become the first-line treatment for a variety of cancers, such as metastatic melanoma [5], metastatic non-small cell lung cancer (NSCLC) [6], head and neck squamous cell carcinoma (HNSCC) [7], metastatic triple-negative breast cancer [8], metastatic renal cell carcinoma (RCC) [9], and hematological malignancies [10]. However, interestingly, anti-PD-1 therapy has shown effectiveness in some PD-L1-negative cancer patients, such as NSCLC and RCC [11,12]. Therefore, the quantification of PD-L1 expression alone may not be comprehensive enough for patient selection in all cases.

PD-L2, as the other crucial ligand of PD-1, is associated with PD-L1 in molecular regulation and mechanism. Previous research has demonstrated that the binding affinity of PD-1 to PD-L2 is approximately 2 to 6 times higher than that of PD-L1 [13], and PD-L2 is highly expressed in a variety of solid tumor tissues, including HNSCC [14], bladder cancer [15], NSCLC [16], RCC [17], and pancreatic ductal adenocarcinoma [18], the elevated expression of PD-L2 is strongly linked to the clinical prognosis of certain tumors following anti-PD-1 treatment, such as, in HNSCC patients, the response rate to anti-PD-1 therapy in both PD-L1- and PD-L2-positive patients was 27.5%, which was higher than the only PD-L1-positive patients (11.4%), it also suggested that PD-L2 status was an important predictor of progression-free survival (PFS) after pembrolizumab treatment, independent of PD-L1, and PD-L2-positive patients had longer median PFS time and overall survival (OS) than PD-L2-negative patients [14]. However, a meta-analysis indicated that PD-L2 overexpression was notably correlated with poorer OS and inferior disease-free survival. Their subgroup analysis further highlighted that increased PD-L2 levels served as an important prognostic marker for worse OS in hepatocellular carcinoma and colorectal cancer [19]. In addition, the high expression of PD-L2 implies a weak tendency to lymphatic metastasis [19]. All these studies confirmed that PD-L2 can be used as an independent predictor of prognosis and clinical response [14,20,21]. When predicting the response to anti-PD-1 immunotherapy, the influence of PD-L2 on anti-PD-1 therapy should also be considered while detecting PD-L1. PD-L2 is expected to be used as a supplementary parameter for further research and application.

Immunohistochemistry (IHC) examination is a commonly used technique for detecting PD-L1 expression in tissue samples. However, IHC has several limitations, particularly in the context of heterogeneous and dynamic monitoring of malignant tumors. As IHC is an invasive examination method, it can be uncomfortable for patients and carries risks such as bleeding, infection, and damage to surrounding tissues. Additionally, IHC provides information about PD-L1 expression in a specific tissue sample taken at a particular time. This limits its ability to capture changes in PD-L1 expression over time or in different areas of the tumor. Due to tumor heterogeneity, a single biopsy may not fully represent the overall PD-L1 expression status of the tumor. Furthermore, biopsy procedures for multiple or distant metastases can be challenging and sometimes impossible due to the location or accessibility of the lesions. As a result, the ability to monitor PD-L1 expression in all tumor sites may be limited. Therefore, the interest in molecular imaging technology for the clinical management of malignant tumors is steadily growing. Molecular imaging in nuclear medicine relies on the use of specific molecular targets and radiopharmaceuticals, which are radionuclide-labeled tracers, to visualize and quantify molecular processes in vivo. These tracers are designed to selectively bind to molecular targets of interest, such as receptors, enzymes, or transporters, allowing for the noninvasive and real-time assessment of molecular expression levels in a particular region of interest within the body [22]. Radiotracers targeting immune checkpoints have garnered influential attention and interest in recent years, particularly in the field of cancer immunotherapy. The molecular probes targeting PD-L1 can be categorized into 3 types based on their molecular structure: Firstly, antibody-based probes, such as [^89^Zr]Zr-nivolumab, are derived from full-length antibodies and are used to target PD-L1 expression, assessing intertumor and intratumor heterogeneity in conditions like NSCLC [23]. Secondly, small-molecule probes are developed from specific small-molecule inhibitors or similar compounds through simple chemical synthesis. For example, the small-molecule positron emission tomography (PET) probe [^18^F]LP-F was used to measure PD-L1 expression in tumors [24]. Thirdly, peptide-based probes, which are derived from short peptide sequences, offer high targeting specificity and low immunogenicity. [^68^Ga]HKP2201 and [^68^Ga]HKP2202, linear peptides with significant potential for the rapid detection of primary and metastatic tumors, including liver cancer [25]. Another example is [^18^F]F-BMS-986229, a macrocyclic peptide ligand targeting PD-L1, which provides improved stability and binding affinity over linear peptides [26,27]. Additionally, the PET imaging tracer [^68^Ga]Ga-AUNP-12, which features a simpler synthesis process, has been used clinically to detect PD-L1 expression in patients with lung and esophageal cancers [28]. In recent years, there have been several previous studies on the use of radionuclide noninvasive imaging to show PD-L2 expression at the animal level, validated by single-photon emission computed tomography or PET imaging, such as ^99m^Tc-PEG6-RD-PDP2 peptide [29], ^124/125^I-labeled mab ATL2 [30], and ^89^Zr-labeled of DFO-conjugated ATL2 [31]. However, the level of PD-L2 expression targeting peptide probe in human has not been reported.

In response to the growing interest in the role of PD-L2 in ICI therapy, we have developed a novel PD-L2 targeting peptide tracer, named HN11-1, which has been labeled with the radionuclide [^68^Ga]Ga. For the first time, we conducted a comprehensive series of experiments encompassing quality control assessments, in vitro and in vivo studies, [^68^Ga]Ga-HN11-1 PET imaging in tumor xenograft models. These investigations were conducted to comprehensively assess the safety and efficacy profile of our tracer. Building upon these findings, we proceeded to validate and assess the clinical imaging efficacy of [^68^Ga]Ga-HN11-1 through pioneering human studies. Additionally, we conducted an evaluation of imaging, biological distribution, radiation dosimetry, and safety outcomes of [^68^Ga]Ga-HN11-1 in both healthy volunteers and patients diagnosed with primary NSCLC and recurrent or metastatic HNSCC. Through these investigations, we aimed to elucidate the clinical utility of our PD-L2 targeting peptide tracer in predicting and assessing the efficacy of ICI therapy, thereby emphasizing its potential value in the realm of immunotherapy.

## Results

### Relationship between PD-L2 expression and clinical response to anti-PD-1 therapy

**Fig. 1.**
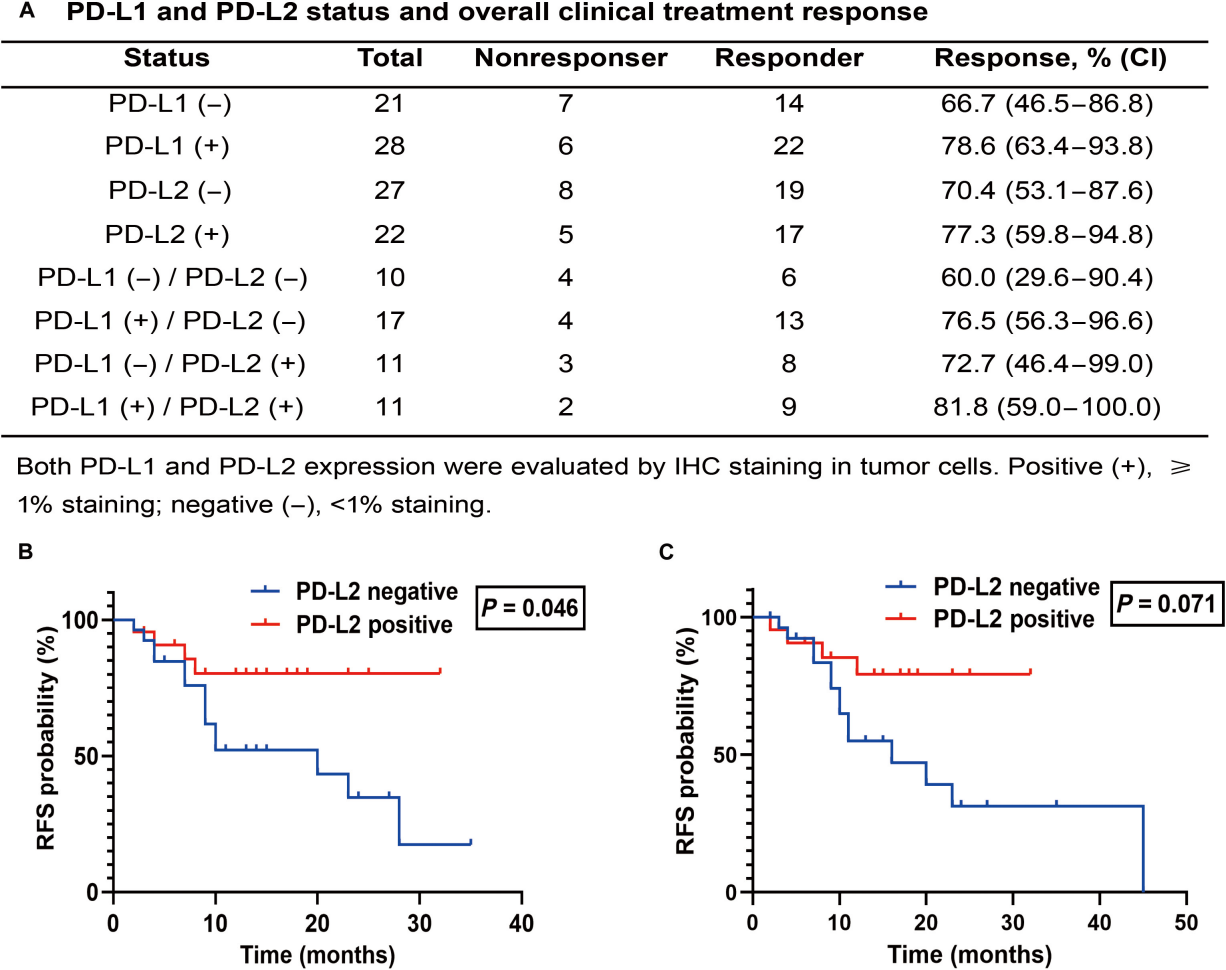
Association between PD-L2 expression and clinical outcomes in response to anti-PD-1 therapy. (A) PD-L1 and PD-L2 status and overall clinical treatment response. K-M curve showing PFS (B) and relapse-free survival (C) probability after treated with pembrolizumab in PD-L2 positive (*n* = 22) group and PD-L2 negative (*n* = 27) group.

We evaluated the clinical relevance between the expression of PD-L2 in tumor tissues and anti-PD-1 immunotherapy (pembrolizumab) in 49 patients with NSCLC, as assessed by PD-L2 IHC score data from these patients. The median age was 60 years (range, 41 to 86 y), male (81.6%). About 57.1% of the patients had distant metastasis (M1 stage), and 42.9% of the patients had received ≥2 other treatment before anti-PD-1 therapy (Table). 28 (57.1%) patients were classified as PD-L1-positive expression, 22 (44.9%) as PD-L2-positive expression, 21 (42.9%) as PD-L1 negative, and 27 (55.1%) as PD-L2 negative (Fig. 1A). The response rates in PD-L1-positive alone (78.6%; 95% confidence interval [CI], 63.4 to 93.8) or PD-L2-positive alone (77.3%; 95% CI, 59.8 to 94.8) were numerically higher than that of both PD-L1 and PD-L2 negative group [60.0%; 95% CI, 29.6 to 90.4] (Fig. 1A). The objective response rate of patients with both PD-L1- and PD-L2-positive [81.8%; 95% CI, 59.0 to 100.0] was higher than in patients with PD-L1-positive while PD-L2 unknown [78.6%; 95% CI, 63.4 to 93.8], and also higher than in patients with PD-L1-positive but PD-L2-negative [76.5%; 95% CI, 56.3 to 96.6] (Fig. 1A). In the 49 patients, PD-L2-positive was identified as a statistically significant predictor of better PFS (*P* < 0.05) (Fig. 1B), but it was not a statistically significant predictor of relapse-free survival (Fig. 1C).

**Table.** Baseline characteristics of patients in NSCLC

Characteristic	Total *N* = 49
Age, median (range), y	60 (41–86)
Gender	
Male	40 (81.6)
Female	9 (18.4)
Metastatic staging	
M0	21 (42.9)
M1	28 (57.1)
Histological type	
Squamous cell carcinoma	12 (24.5)
Adenocarcinoma	37 (75.5)
Previous other adjuvant therapy ^a^	
Yes	41 (83.7)
No	8 (16.3)
No. of previous lines of other adjuvant therapy	
0	8 (16.3)
1	19 (38.8)
2	21 (42.9)
3	1 (2.0)

^a^ Other adjuvant therapies include chemotherapy, radiation, surgery, and targeted therapy.

### Characterization and radiochemistry

**Fig. 2.**
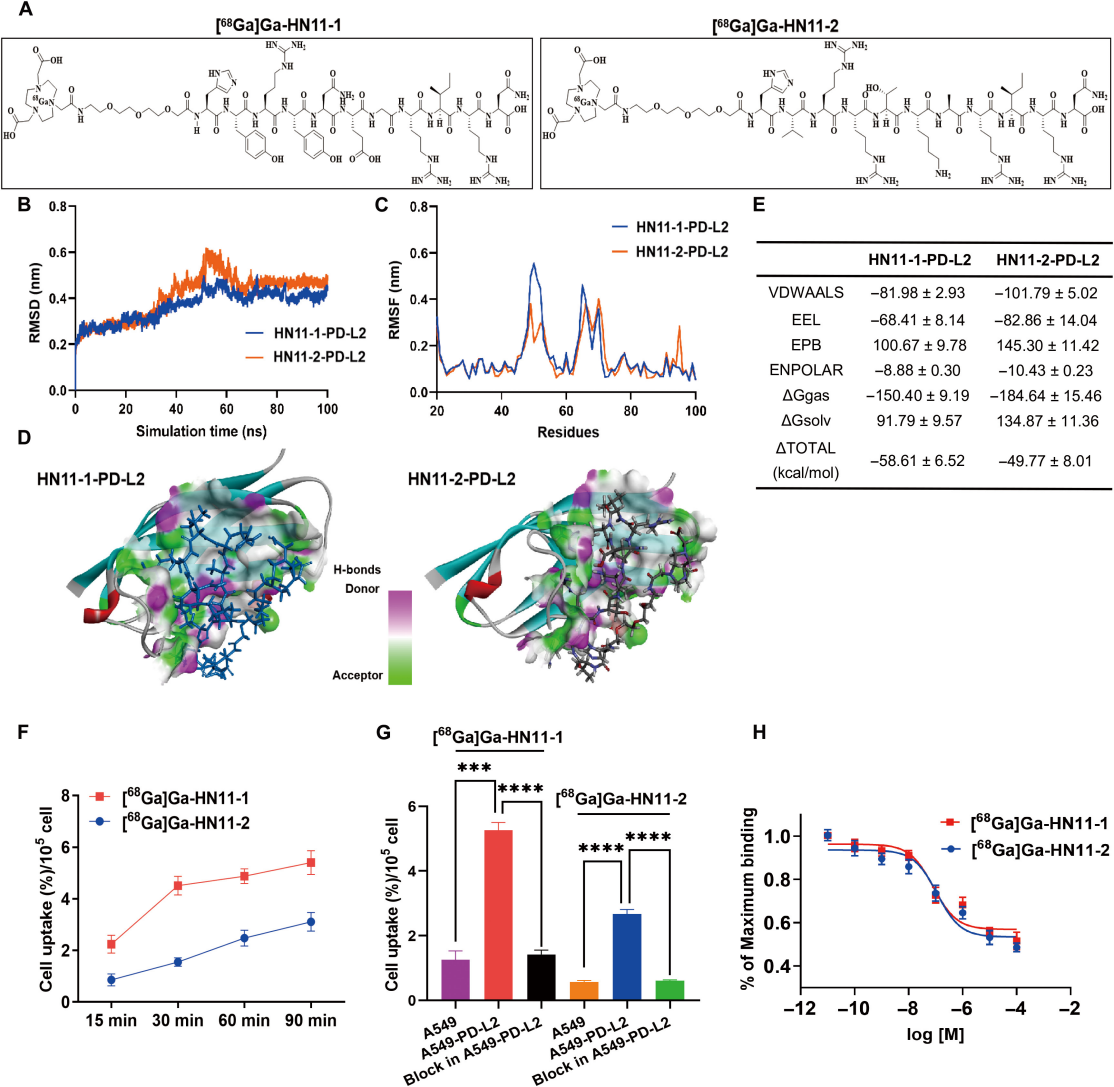
Characterization, radiochemistry, and in vitro validation. (A) The chemical structures of [^68^Ga]Ga-HN11-1 and [^68^Ga]Ga-HN11-2. (B) The root mean square deviation of the backbone atoms relative to their initial minimized complex structures as a function of time. (C) The root mean square fluctuation of the backbone atoms (CA, N, C) versus residue number for the HN11-1–PD-L2 and HN11-2–PD-L2 compared to the initial structures. (D) The 3D contour of HN11-1 and HN11-2 interacted to PD-L2, the solvent accessible surface (SAS) of binding pocket was colored by the hydrogen bond receptor donor. (E) Binding free energies and individual energy terms of HN11-1 and HN11-2 in complex with DDR1. (F) Cellular uptakes of [^68^Ga]Ga-HN11-1 and [^68^Ga]Ga-HN11-2 in the A549-PD-L2 cell lines after 15, 30, 60, and 90 min of incubation (*n* = 5). (G) Cell uptakes of [^68^Ga]Ga-HN11-1, [^68^Ga]Ga-HN11-2 in A549 and A549-PD-L2 cell lines and their block group in A549-PD-L2 cell lines after 60 min of incubation(*n* = 5). (H) IC_50_ of [^68^Ga]Ga-HN11-1 and [^68^Ga]Ga-HN11-2. The data are shown as mean ± SD, ****P < 0.0001, ***P < 0.001, **P < 0.01, *P < 0.05.

We have designed 2 specific peptide antagonists targeting PD-L2, which were HN11-1 and HN11-2, respectively (Fig. S1A and B). The high-performance liquid chromatography of HN11-1and HN11-2 were shown in Fig. S1C and D. The chemical structure of the [^68^Ga]Ga-HN11-1/[^68^Ga]Ga-HN11-2 is shown in Fig. 2A. The overall radiochemical yield of [^68^Ga]Ga-HN11-1 and [^68^Ga]Ga-HN11-2 were 77.3 ± 7.2% and 74.5 ± 4.4%, respectively (*n* = 5), radiochemical purity of both were >99% (*n* = 5), the molar activity were 75.3 ± 0.17 GBq/μmol and 36.3 ± 0.32 GBq/μmol (*n* = 5), and the partition coefficients (log *P*) at pH 7.4 were −0.30 ± 0.0017 and −0.27 ± 0.0018 (*n* = 5), respectively, indicating that both radiotracers were hydrophilic (Fig. S2). These precursors were radiolabeled with ^68^GaCl_3_ and subsequently purified using C_18_ solid-phase extraction cartridges, with verification performed using radio-high-performance liquid chromatography in 0.5 and 4 h (Fig. S3). The excellent in vitro saline and serum stability of both radiotracers were > 95% over 4 h at room temperature (Fig. S4).

### The binding modes by molecular dynamics simulation

As shown in Fig. 2B to E, the potential molecular binding mode of HN11-1 and HN11-2 to the kinase domain of PD-L2 were employed by molecular dynamics simulations. The reliability of the MD simulations for both HN11-1-PD-L2 and HN11-2-PD-L2 complexes was confirmed by the root mean square deviation values, which was showed in Fig. 2B. The binding modes and compute binding free energy were analyzed by the stable simulation trajectory. The root mean square fluctuation distributions of HN11-1-PD-L2 and HN11-2-PD-L2 complexes were comparable to the crystallized PD-L2 protein (Fig. 2C). As shown in Fig. 2D, hydrogen bonds and potential halogen bonds were formed with some groups. The binding free energy and energy decomposition of HN11-1-PD-L2 and HN11-2-PD-L2 complexes were calculated by the molecular mechanics/Poisson–Boltzmann surface area approach and intermolecular van der Waals, surface area interactions, and electrostatic interactions were demonstrated as the main contributors to ligand binding (Fig. 2E).

### Cell uptake and binding assays

We successfully transfected PD-L2 into the A549 cell line using transfection techniques, creating the A459-PD-L2 cell line. Verification of transfection was achieved through Western blot and real-time quantitative polymerase chain reaction, respectively (Fig. S5A and B). Figure 2F shows that at 30 min, the cell uptake rate of [^68^Ga]Ga-HN11-1 by A549-PD-L2 cells was 4.51 ± 0.04%, which was 3 times that of [^68^Ga]Ga-HN11-2 (1.55 ± 0.08%) (*P* < 0.001, *n* = 5). At 60 min, the cell uptake rate of [^68^Ga]Ga-HN11-1 by A549-PD-L2 cells was still nearly twice that of [^68^Ga]Ga-HN11-2 cells, which were 4.87 ± 0.28% and 2.47 ± 0.13%, respectively (*P* < 0.001, *n* = 5), but in [^68^Ga]Ga-HN11-1 + block and [^68^Ga]Ga-HN11-2 + block groups, cell uptake were markedly decreased to 1.43 ± 0.13% and 0.61 ± 0.03% (*P* < 0.001, *n* = 5) at 60 min after pretreatment with excess of HN11-1 and HN11-2 (Fig. 2G). The half-maximal inhibitory concentration values of 81.4 nM for [^68^Ga]Ga-HN11-1 and 113.2 nM for [^68^Ga]Ga-HN11-2 indicate that both radiotracers have high affinity (Fig. 2H), and surface plasmon resonance of HN11-1 was 8.49 × 10^−8^ (Fig. S6).

### Dynamic MicroPET imaging, pharmacokinetics, and biodistribution

**Fig. 3.**
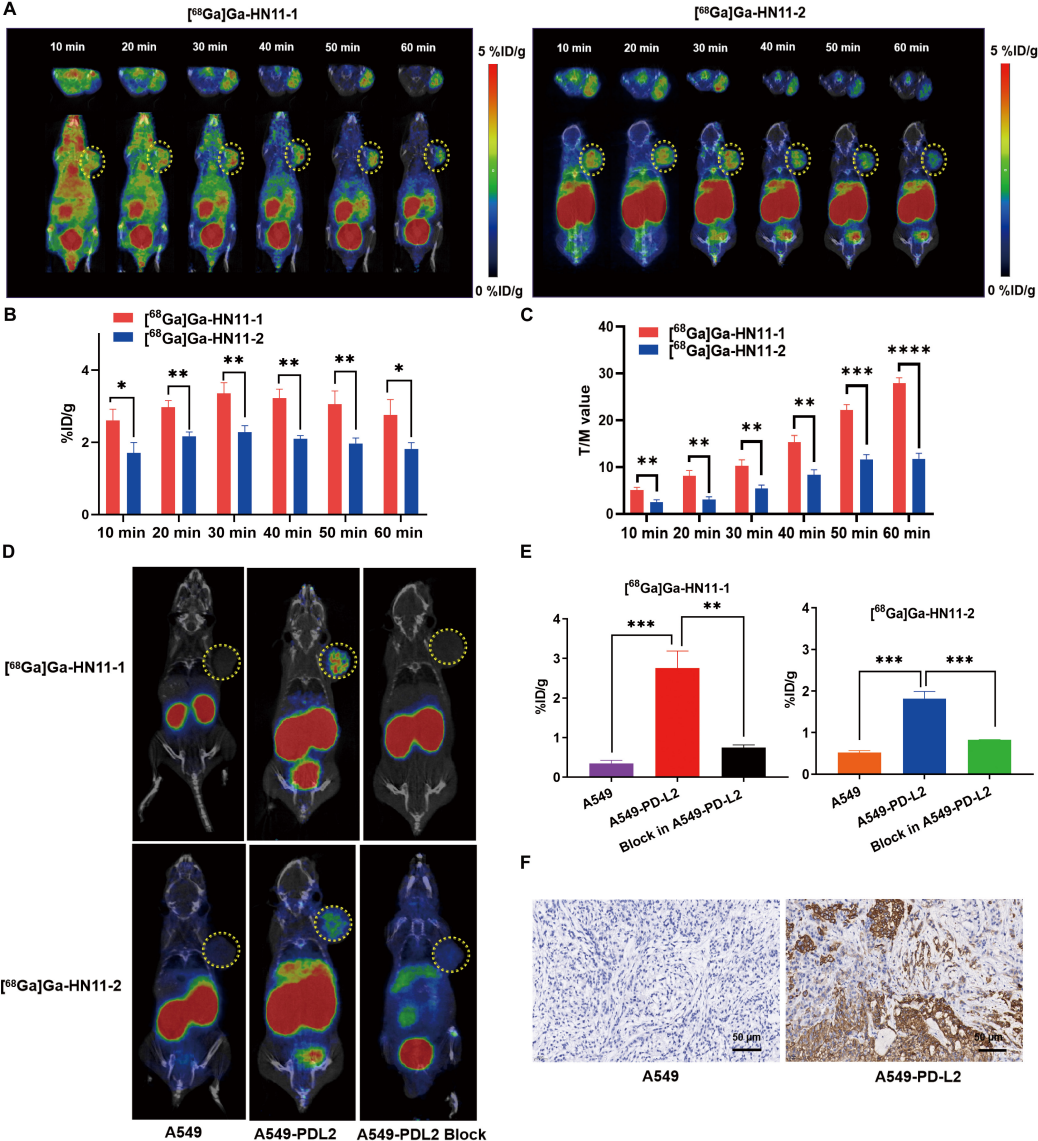
In vivo validation and IHC. (A) Dynamic PET imaging in A549-PD-L2 xenograft models after injection of [^68^Ga]Ga-HN11-1, [^68^Ga]Ga-HN11- 2 at 10, 20, 30, 40, 50, and 60 min (*n* = 3). (B) The tumor uptakes of [^68^Ga]Ga-HN11-1, [^68^Ga]Ga-HN11-2 (%ID/g) in the A549-PD-L2 xenograft models at 10, 20, 30, 40, 50, and 60 min (*n* = 3). (C) The T/M ratios of [^68^Ga]Ga-HN11-1, [^68^Ga]Ga-HN11-2 in A549-PD-L2 xenograft models at 10, 20, 30, 40, 50, and 60 min (*n* = 3). (D) PET/CT imaging at 60 min in A549 and A549-PD-L2 xenograft models after injection of [^68^Ga]Ga-HN11-1, [^68^Ga]Ga-HN11-2, and their block group in A549-PD-L2 xenograft models(*n* = 3). (E) The quantitative data of [^68^Ga]Ga-HN11-1, [^68^Ga]Ga-HN11-2, [^68^Ga]Ga-HN11-1 + block, and [^68^Ga]Ga-HN11-2 + block illustrate the tumor uptake fraction (%ID/g). (F) PD-L2 IHC staining of A549 and A549-PD-L2 tumor tissues (*n* = 3, scale = 50 μm).

Figure 3A and Fig. S6A show dynamic PET imaging of [^68^Ga]Ga-HN11-1, [^68^Ga]Ga-HN11-2 in A459-PD-L2 tumor xenograft models, and [^68^Ga]Ga-HN11-1 in A549 tumor xenograft models at 10, 20, 30, 40, 50, and 60 min after injection. At each time point, the tumor uptake values of [^68^Ga]Ga-HN11-1 were 2.61 ± 0.16%, 2.97 ± 0.18%, 3.36 ± 0.29%, 3.22 ± 0.23%, 3.06 ± 0.36%, and 2.76 ± 0.42%ID/g in A459-PD-L2 tumor xenograft models, respectively; these values notably exceed those observed in the [^68^Ga]Ga-HN11-2 group, which were 1.71 ± 0.14%, 2.17 ± 0.12%, 2.29 ± 0.17%, 2.10 ± 0.03%, 1.97 ± 0.07%, and 1.82 ± 0.06%ID/g, respectively (Fig. 3B). However, in the A549 tumor xenograft models, the tumor uptakes of [^68^Ga]Ga-HN11-1 was almost negligible at each time point (Fig. S7A). The T/M ratios in [^68^Ga]Ga-HN11-1 and [^68^Ga]Ga-HN11-2 groups in the A549-PD-L2 tumor xenograft models were 5.08 ± 0.84, 10.31 ± 2.18, 27.92 ± 1.76, and 2.60 ± 0.60, 5.44 ± 1.12, and 11.81 ± 1.93 at 10, 30, and 60 min, respectively (Fig. 3C), both groups exhibited T/M ratios higher than those observed in the A549 tumor xenograft models after injection of [^68^Ga]Ga-HN11-1 to varying degrees (Fig. S7B). The pharmacokinetics study revealed that [^68^Ga]Ga-HN11-1 was more rapidly cleared from the blood than [^68^Ga]Ga-HN11-2, the half-life were 13.37 and 22.66 min, respectively (Fig. S8).

The biodistribution of [^68^Ga]Ga-HN11-1 was performed in a successful A549-PD-L2 xenograft tumor models at 10, 30, 60, and 90 min postinjection. The uptake of [^68^Ga]Ga-HN11-1 was highest in the kidney compared to nonspecific organs like the heart, liver, and lung. Optimal tumor-to-organ ratios were observed 60 min postinjection, calculated as follows: 12.72 ± 2.22 for Tumor/Heart, 2.53 ± 0.75 for Tumor/Liver, 4.11 ± 1.25 for Tumor/Muscle, and 6.41 ± 0.98 for Tumor/Blood (Fig. S9 and Table S1).

### Verification through in vivo and IHC methods

Compared with A549 xenograft tumor models, [^68^Ga]Ga-HN11-1, [^68^Ga]Ga-HN11-2 could markedly accumulate in the tumor sites of A549-PD-L2 xenograft tumor models at 60 min in Fig. 3D (*n* = 3), after blocking with excess of HN11-1 and HN11-2, tumor uptakes of [^68^Ga]Ga-HN11-1 and [^68^Ga]Ga-HN11-2 were decreased to 0.74 ± 0.03% and 0.82 ± 0.04%ID/g at 60 min, respectively (Fig. 3E). After the imaging, the tumor tissues in A549 and A459-PD-L2 xenograft tumor models were removed, and the expression of PD-L2 was tested by IHC, which verified the xenograft tumor model was successfully constructed (Fig. 3F). After thorough comparison, it was determined that [^68^Ga]Ga-HN11-1 exhibited superior characteristics compared to [^68^Ga]Ga-HN11-2 across various parameters including cell uptake capacity, T/M ratio, and micro-PET imaging in A549-PD-L2 xenograft models. As a result, [^68^Ga]Ga-HN11-1 was selected as the preferred tracer for subsequent human experiments.

### Human organ distribution and dosimetry

Between December 2021 and April 2023, 3 healthy volunteers and 6 patients (2 primary NSCLC patients, 2 local recurrent HNSCC patients, 1 bone metastasis HNSCC patient and 1 lung metastasis HNSCC patient) received the intravenous injection of [^68^Ga]Ga-HN11-1 (3.1 to 4.9 MBq/kg), with well tolerated and without adverse pharmacological reactions observed.

**Fig. 4.**
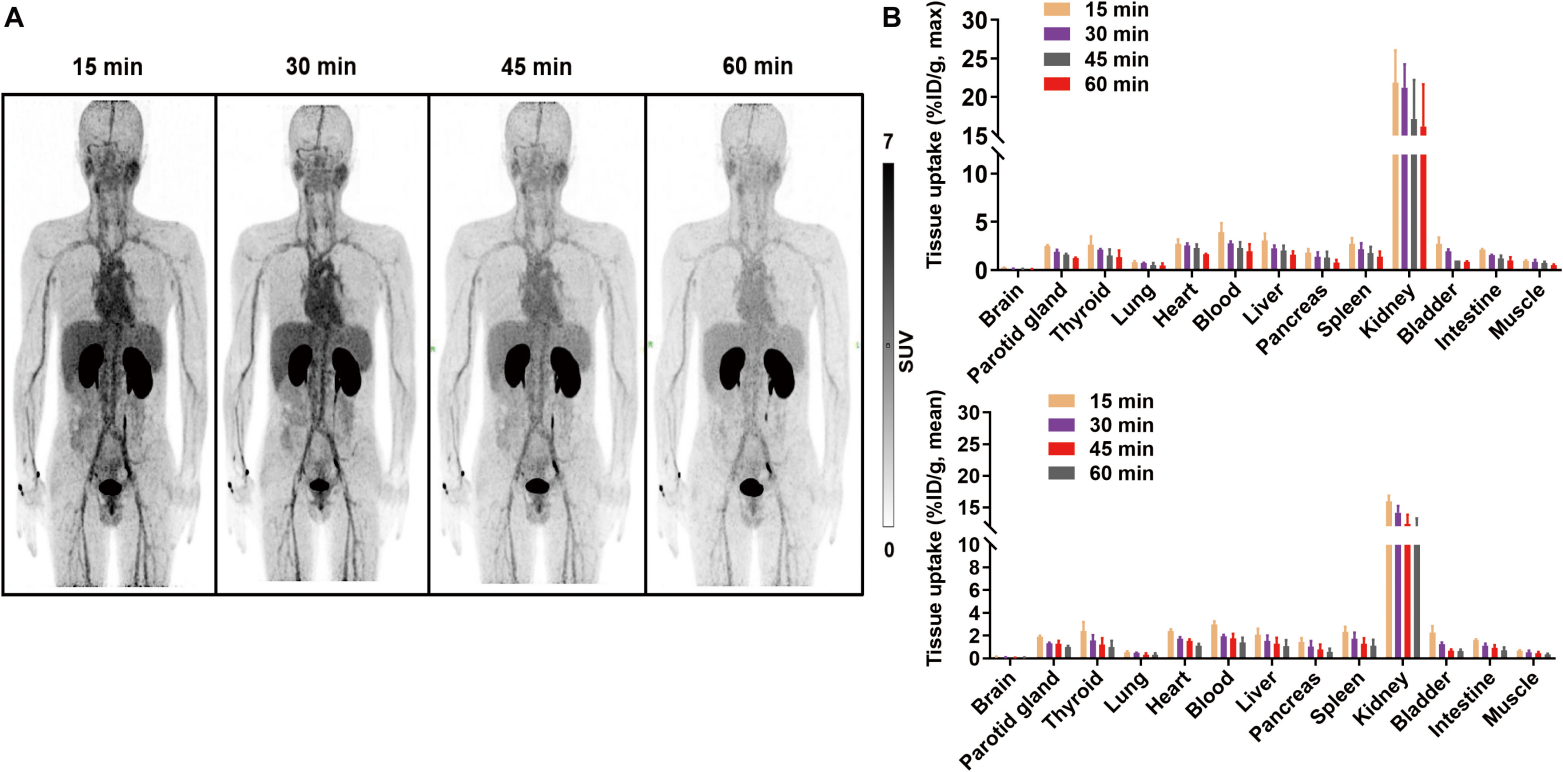
[^68^Ga]Ga-HN11-1 PET imaging in healthy volunteers (*n* = 3). (A) Maximum intensity projection imaging at different time points after injection. (B) The SUV_max_ and SUV_mean_ values of normal organ uptakes of [^68^Ga]Ga-HN11-1.

As shown in Fig. 4A, [^68^Ga]Ga-HN11-1 exhibits the highest distribution in the urinary system and is rapidly excreted over time. Additionally, it moderately accumulates in the heart, liver, blood, and spleen, with lower uptake observed in the brain, thyroid, and muscle. Quantified values of SUV_max_ and SUV_mean_ in each organ are depicted in Fig. 4B.

Table S2 shows the specific radiation dose distribution data (SUV_mean_) for healthy volunteers. The kidney received the highest doses (9.83 × 10^−2^ ± 2.10 × 10^−2^ mSv/MBq). Additionally, the effective dose, measured at 5.79 × 10^−3^ ± 1.25 × 10^−3^ mSv/MBq, is lower than using [^18^F]F-FDG PET/CT (Adult 1.9 × 10^−2^ mSv/MBq, CT: computed tomography) [31].

### [^68^Ga]Ga-HN11-1 PET/CT imaging in various cancer patients

**Fig. 5.**
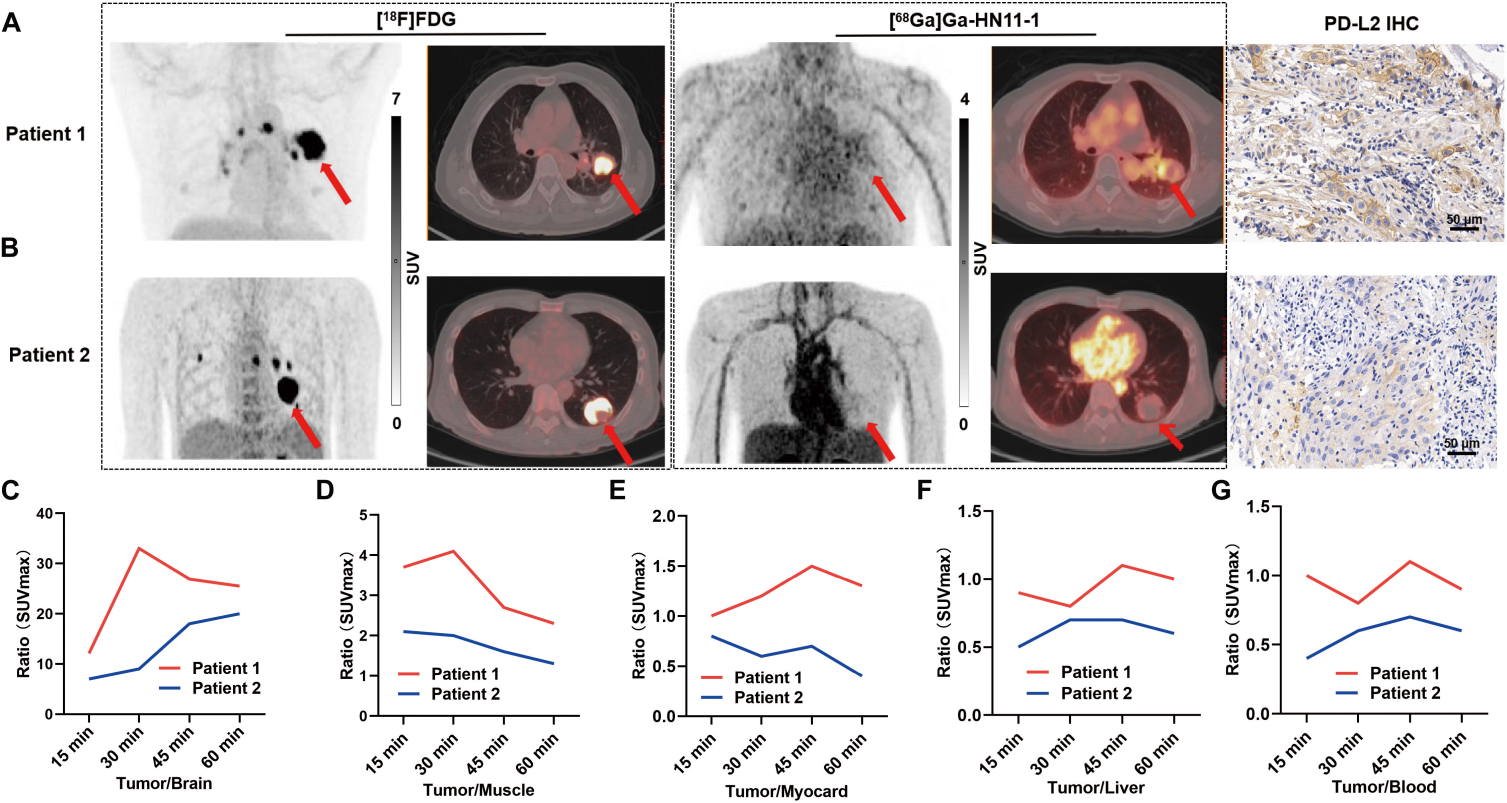
[^68^Ga]Ga-HN11-1 and [^18^F]F-FDG PET/CT imaging in primary lung adenocarcinoma patients (tumor location is indicated by the red arrow). (A) Maximum Intensity Projection (MIP) imaging, transverse imaging, coronal imaging, and PD-L2 high expression of patient 1. (B) MIP imaging, transverse imaging, coronal imaging, and PD-L2 low expression of patient 2. The SUV_max_ of [^68^Ga]Ga-HN11-1 in tumor/other organs (%) between patient 1 and patient 2. (C) Tumor/Brain. (D) Tumor/Muscle. (E) Tumor/Myocard. (F) Tumor/Liver. (G) Tumor/Blood.

Six patients (1 female and 5 male; median age: 59.5 y, range: 41-68 y), all patients accepted [^68^Ga]Ga-HN11-1 PET/CT scan after [^18^F]F-FDG completed PET/CT scan within 5 d. The mean administered dose of [^68^Ga]Ga-HN11-1 was 248 ± 22 MBq (range, 222 to 312 MBq). As shown in Fig. 5A and B, the 2 patients with lung adenocarcinoma had different expressions of PD-L2, high PD-L2 expression resulted in higher tumor uptake of [^68^Ga]Ga-HN11-1 in patient 1 (IHC score 80%, SUV_max_: 4.1, Fig. 5A) compared to patient 2, who had low PD-L2 expression (IHC score 15%, SUV_max_: 2.1, Fig. 5B), while the SUV_max_ of [^18^F]F-FDG was 15.5 for patient 1 and 25.9 for patient 2. Thus, the uptake of [^68^Ga]Ga-HN11-1 correlated with high PD-L2 expression, while the uptake of [^18^F]F-FDG was not related to PD-L2 status. Additionally, we also compared the SUV_max_ of [^68^Ga]Ga-HN11-1 in tumor/other organs (%) between patient 1 and patient 2, as shown in Fig. 5C to G, which could provide valuable insights into the tracer’s specificity and distribution patterns.

**Fig. 6.**
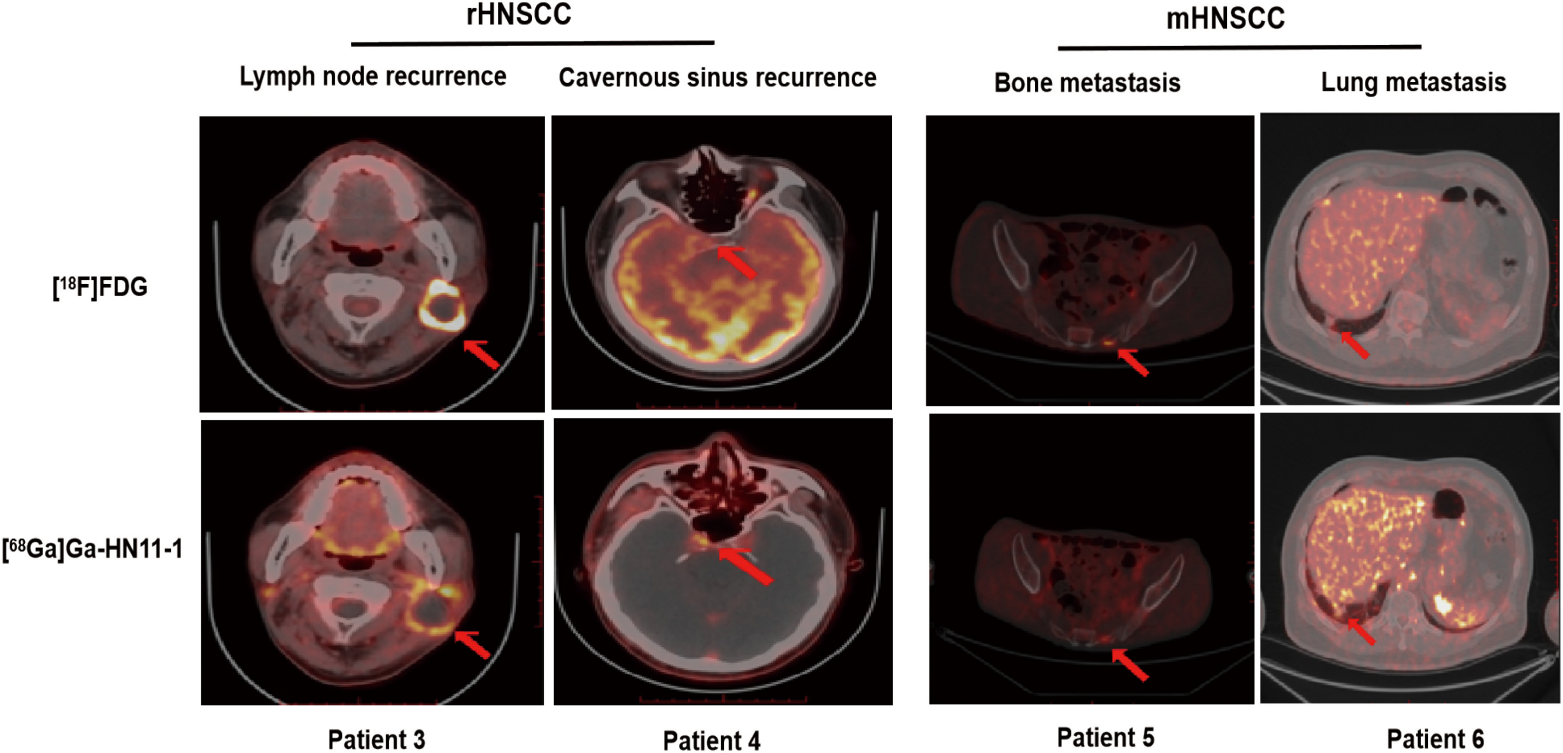
[^68^Ga]Ga-HN11-1 and [^18^F]F-FDG PET/CT imaging in recurrent and metastatic HNSCC patients (tumor location is indicated by the red arrow). Patient 3 and patient 4 were transverse imaging for recurrent HNSCC. Patient 5 and patient 6 were transverse imaging for bone and lung metastases of HNSCC.

Figure 6 presents 4 cases of recurrent or metastatic HNSCC, all of which were identified as squamous cell carcinoma. Patient 3 developed left cervical lymph node recurrence following buccal cancer surgery. In this patient, the increased [^68^Ga]Ga-HN11-1 metabolism area was similar to that observed with [^18^F]F-FDG, and liquefaction necrosis was present in the recurrent lymph nodes. The SUV_max_ values for [^68^Ga]Ga-HN11-1 and [^18^F]F-FDG were measured as 4.0 and 13.7, respectively. Patient 4 presented with residual postoperative invasion of the cavernous sinus of the skull base, [^68^Ga]Ga-HN11-1 PET imaging provided clearer visualization of the lesion compared to [^18^F]F-FDG. This enhanced clarity is attributed to the reduced interference of [^68^Ga]Ga-HN11-1 with brain metabolism, facilitating better delineation of the lesion. The SUV_max_ values for the lesion were measured as 3.0 with [^68^Ga]Ga-HN11-1 and 4.0 with [^18^F]F-FDG. Patient 5 had tongue cancer with bone metastasis after surgery, increased [^18^F]F-FDG metabolism was detected in the left side of the sacrum, there was a slight increase in [^68^Ga]Ga-HN11-1 uptake in this area, suggesting potential PD-L2 expression in the metastatic lesion. Patient 6 presented with lung metastasis following surgery for laryngeal cancer, both [^68^Ga]Ga-HN11-1 and [^18^F]F-FDG metabolism exhibited a similar extent in the lung metastasis. Table S3 summarized all patient characteristics, PD-L2 expression and SUV_max_ of [^18^F]F-FDG and [^68^Ga]Ga-HN11-1. The median SUV_max_ of [^68^Ga]Ga-HN11-1 PET were 3.25 (range, 2.1 to 4.1) (*n* = 6). The median SUV_max_ of [^18^F]F-FDG were 11.1 (range, 3.7 to 25.9) (*n* = 6).

## Discussion

With the widespread adoption of targeted monoclonal antibodies in clinical practice, PD-L1 status has traditionally been utilized as a key screening criterion for immunotherapy [1–3,32]. However, as research progresses, it has become evident that relying solely on PD-L1 expression may not provide a comprehensive assessment for patient selection. With the negative PD-L1 expression still benefit from immunotherapy, suggesting the need for additional biomarkers [11,12]. Several studies have highlighted PD-L2 expression, either alone or in combination with PD-L1, as an important biomarker for predicting the prognosis of immunotherapy across various tumor types [14,33]. High PD-L2 expression has been associated with a favorable prognosis in multiple tumor immunotherapy settings [14]. In the context of NSCLC, our study has demonstrated that high PD-L2 expression is correlated with a better prognosis in terms of PFS for NSCLC patients undergoing anti-PD-1 therapy.

Indeed, while there has been growing interest in developing radiotracers targeting PD-L2 for potential clinical applications, most research in this area is still at the preclinical stage, primarily involving animal studies [29–31]. These studies aim to evaluate the feasibility, specificity, and efficacy of PD-L2-targeted radiotracers in preclinical models of cancer. [^89^Zr]Zr-DFO-ATL2 is a promising radiotracers in terms of effectively displaying PD-L2 expression in tumor tissue with relatively low injection doses and high SUV_max_ values, which injection dose is only 1.85 MBq, but the SUV_max_ value of tumor tissue detection can be as high as 3.53 ± 0.09. The successful demonstration of [^89^Zr]Zr-DFO-ATL2 in effectively displaying PD-L2 expression in tumor tissue marks a substantial advancement in the field of molecular imaging. However, there are several challenges associated with its clinical application; firstly, due to the large molecular weight of [^89^Zr]Zr-DFO-ATL2, it exhibits a prolonged distribution time in the human body. This necessitates a longer imaging window, typically around 5 to 7 d postinjection, to allow for optimal visualization of PD-L2 expression in tumor tissue. In addition, the production process of [^89^Zr]Zr-DFO-ATL2 is relatively complex, posing challenges in terms of manufacturing, quality control, and scalability for widespread clinical use [31]. As for [^124^I]I-ATL2, although it can effectively display PD-L2 expression in tumor tissues, there are specific challenges associated with its clinical application. Firstly, the iodine component in [^124^I]I-ATL2 can accumulate in the thyroid gland, leading to potential interference with thyroid imaging and potential radiation exposure to the thyroid. Secondly, [^124^I]I-ATL2 may not be suitable for detecting tumors in certain anatomical locations, such as the gastric system and urinary system, this limitation could restrict its utility in detecting malignancies arising from these organs. Overall, while [^124^I]I-ATL2 holds promise as a tracer for PD-L2 imaging, its limitations may hinder its clinical transformation and widespread adoption [30]. In this study, we constructed a new radionuclide molecular tracer, [^68^Ga]Ga-HN11-1 as a radionuclide molecular tracer targeting PD-L2 represents a substantial advancement in cancer imaging and therapy. This tracer, being a small-molecule peptide compound, offers several advantages. Firstly, [^68^Ga]Ga-HN11-1 is designed to specifically target and bind to PD-L2, enabling accurate visualization and quantification of PD-L2 expression in tumor tissues. Secondly, the small molecular weight of [^68^Ga]Ga-HN11-1 facilitates rapid diffusion and penetration into tumor tissues, leading to efficient accumulation within the tumor microenvironment. This characteristic enhances the tracer’s ability to delineate tumor boundaries and detect small lesions. Thirdly, we have demonstrated that [^68^Ga]Ga-HN11-1 effectively accumulates in tumor tissues in vivo and in vitro studies, particularly in A549-PD-L2 xenograft tumors, the T/M ratio of [^68^Ga]Ga-HN11-1 in A549-PD-L2 tumor xenograft model at 60 min after injection is reported as 27.92 ± 1.76. This robust tumor accumulation is crucial for achieving high imaging sensitivity and contrast. Finally, the favorable pharmacokinetic profile of [^68^Ga]Ga-HN11-1, characterized by rapid clearance from nontarget tissues and organs, contributes to its low systemic toxicity and excellent safety profile in preclinical studies.

The validation test conducted in human subjects represents a major milestone in the development of [^68^Ga]Ga-HN11-1 as a molecular imaging tracer targeting PD-L2. The dynamic observation of PD-L2 expression changes using immune PET imaging in healthy volunteers and cancer patients provides valuable insights into the tracer’s pharmacokinetics, biodistribution, and safety profile in humans. Our study at first time demonstrates that the radiation dose distribution of [^68^Ga]Ga-HN11-1 in the human body is safe, indicating acceptable levels of radiation exposure for patients undergoing imaging with this tracer, and the tracer is primarily excreted through the kidneys, suggesting a renal clearance pathway. This rapid renal excretion contributes to the tracer’s favorable pharmacokinetic profile and minimizes systemic exposure, reducing the risk of toxicity. Additionally, [^68^Ga]Ga-HN11-1 exhibits a short half-life of only 13.37 min, indicating rapid decay and clearance from the body. This short half-life enables timely imaging with high contrast and minimizes radiation exposure to patients and medical staff. Most importantly, the tracer demonstrates effective enrichment in tumor tissues expressing PD-L2, with its expression level correlating with the degree of tumor uptake. The observation that patient 1, with high PD-L2 expression, exhibited higher tumor uptake of [^68^Ga]Ga-HN11-1 compared to patient 2, this suggests that the [^68^Ga]Ga-HN11-1 might reflect different aspects of tumor biology. Apart from detecting primary tumors, the detection ability of [^68^Ga]Ga-HN11-1 compared to [^18^F]F-FDG in patients with recurrent and metastasis HNSCC represents a substantial advancement in cancer imaging, particularly in challenging anatomical locations such as lesions invading the cavernous sinus close to brain tissue in patient 4. Overall, the validation test in human subjects provides strong evidence supporting the clinical translation of [^68^Ga]Ga-HN11-1 as a safe and effective molecular imaging tracer for PD-L2 expression in cancer patients.

Despite the promising findings, our study has several limitations that warrant consideration. One notable limitation is the small sample size of patients included in the pilot human study. Furthermore, the study design may not have accounted for all potential confounding factors that could influence PD-L2 expression or treatment response. Future studies should consider incorporating comprehensive clinical and molecular profiling to better understand the underlying mechanisms and predictors of response to PD-L2 immunotherapy. Lastly, as with any novel imaging agent, further optimization of [^68^Ga]Ga-HN11-1 synthesis, imaging protocols, and interpretation criteria may be necessary to maximize its clinical utility and standardization across different clinical settings. Addressing these limitations through well-designed prospective studies with larger sample sizes and longer follow-up periods will be critical for advancing our understanding of [^68^Ga]Ga-HN11-1 PET/CT and its role in guiding immunotherapy strategies for cancer patients.

In the work, we have successful developed a novel small-molecule peptide probe [^68^Ga]Ga-HN11-1 for evaluating PD-L2 status in different cancer types via PET imaging. Our findings demonstrate that [^68^Ga]Ga-HN11-1 exhibits high affinity, specificity, decent pharmacokinetics, and robust imaging capabilities, making it a promising PET tracer for evaluating tumor PD-L2 expression. Moreover, the results from our initial human trial are encouraging, confirming the potential clinical utility of [^68^Ga]Ga-HN11-1 PET/CT as a valuable tool for predicting and assessing the efficacy of PD-L2 immunotherapy. This represents a significant step forward in the development of immunoimaging technology, offering clinicians a noninvasive and dynamic approach to monitor PD-L2 expression and response to immunotherapy in cancer patients. Moving forward, our findings provide a solid foundation for further clinical research and the continued advancement of immunoimaging technology. The integration of [^68^Ga]Ga-HN11-1 PET/CT into routine clinical practice has the potential to improve patient outcomes by enabling more personalized treatment strategies and facilitating early intervention in cancer management. Overall, our study underscores the importance of immunoimaging in advancing precision medicine and guiding the development of novel immunotherapeutic approaches for cancer treatment.

## Materials and Methods

### Construction peptide fragments and labeling

We constructed 2 peptide fragments with similar structure to PD-L2, named HN11-1 (peptide sequence: HYRYNEGRIRN) and HN11-2 (peptide sequence: HVRRTKARIRN), respectively. Both of them were synthesized according to custom specifications by GL Biochem (Shanghai, China) Ltd. ^68^Ga was produced using the ^68^Ga/^68^Ge generator stationed at the PET Center Department, Xiangya Hospital, Central South University. Further chemicals and equipment information in this study is shown in the Supporting Information (SI).

### Cell culture, cell transfection, and xenograft models

We chose the A549 cell line along with its PD-L2 transfected counterpart (A459-PD-L2) for conducting both in vitro and in vivo studies. For detailed information on cell culture, cell transfection, and the creation of xenograft models, please refer to the SI.

### Cell uptake and binding analysis

Cell uptake assays were conducted to measure the uptake of [^68^Ga]Ga-HN11-1 and [^68^Ga]Ga-HN11-2 in A549 and A459-PD-L2 cell lines following incubation periods of 15, 30, 60, and 90 min. Blocking studies were performed by coincubating cells with an excess of precursor (HN11-1 / HN11-2), and then the cell uptakes of [^68^Ga]Ga-HN11-1 and [^68^Ga]Ga-HN11-2 were performed in A459-PD-L2 cell lines after 60 min of incubation. The experiment details are provided in the SI.

### Dynamic PET imaging and biodistribution

Micro-PET imaging was conducted using an Inveon PET scanner (Siemens, Germany). Dynamic PET examine were acquired over the first 60 min following the simultaneous injection of 7.4 to 9.25 MBq (200 to 250 μCi) of [^68^Ga]Ga-HN11-1 and [^68^Ga]Ga-HN11-2. After reconstructing the images and delineating the regions of interest (ROIs), time-radioactivity curves were plotted to obtain the Tumor/Muscle (T/M) ratios. %ID/g represents the percentage of the injected dose of the radiotracer that accumulates in a specific tissue per gram of tissue weight. For the biodistribution study, the xenograft models received an intravenous injection of 3.7 to 5.5 MBq (100 to 150 μCi) of [^68^Ga]Ga-HN11-1. After the designated period (10, 30, 60, and 90 min), the mice were euthanized, and important tissues and organs were dissected and collected. The samples were counted using the γ-counter for determining the %ID/g, presented as mean ± standard deviation (SD).

### In vivo verification and pharmacokinetics

The static PET/CT imaging in A549 and A549-PD-L2 xenograft models after injection of 5.55 to 7.4 MBq (150 to 200 μCi) of [^68^Ga]Ga-HN11-1 or [^68^Ga]Ga-HN11-2 were acquired at 60 min. In the [^68^Ga]Ga-HN11-1 + block or [^68^Ga]Ga-HN11-2 + block groups, the xenograft models underwent pretreatment via intravenous injection of HN11-1 or HN-2 (10 mg/kg) before PET/CT imaging. Then, ROIs were delineated for each group. After imaging, tumor tissues from both the A549 and A459-PD-L2 xenograft tumors were extracted, and the PD-L2 expression was tested by IHC. For the pharmacokinetics study, healthy BALB/C mouse received an intravenous injection of 5.55 MBq (150 μCi) of [^68^Ga]Ga-HN11-1 and [^68^Ga]Ga-HN11-2. Measure the activity of blood samples (%ID/g, mean ± SD) at the corresponding time points, and the time-activity curve was plotted. Subsequently, pharmacokinetic parameters were tested using the noncompartmental model in Phoenix WinNonlin 8.1 software (Certara, USA).

### PET imaging in humans

The program content was explained to all healthy volunteers and cancer patients, and written informed consent was obtained. Whole-body dynamic imaging was conducted for 60 min using a PET/CT 690 Elite scanner (GE Discovery) immediately after intravenous injection of [^68^Ga]Ga-HN11-1 (3.1 to 4.9 MBq/kg). A static body scan was performed 15 to 45 min after injection. All cancer patients underwent [^18^F]F-FDG and [^68^Ga]Ga-HN11-1 PET/CT imaging within 5 d. Two nuclear medicine physicians reconstructed and reviewed the images on GE aw 4.6 workstations and reached a consensus. They delineated the ROIs and measured the values of maximum standard uptake and average standard uptake (SUV_max_ and SUV_mean_). The biological distribution of [^68^Ga]Ga-HN11-1 in healthy volunteers was determined by SUV_max_ and SUV_mean_. The OLINDA/EXM software (version 1.0, Hermes Medical Solutions AB) was used to estimate the human radiation dose based on the SUV_mean_ of each organ [34].

### Statistical analysis

Using GraphPad Prism 8.0.2 software. Survival curves were calculated using Kaplan-Meier (K-M) estimates, and differences between groups were assessed using the log-rank test. All data were expressed as mean ± SD, and statistical significance was defined as *P < 0.05, **P < 0.01, ***P < 0.001, ****P < 0.0001.

## Data Availability

The data that support the findings of this study are available from the corresponding author upon reasonable request.
