# Supplementary material for: Noninvasive Monitoring of Programmed Death-Ligand 2 Expression with Positron Emission Tomography using 68Ga-labeled Peptide Antagonist in Preclinical and Exploratory Human Studies
Source: Research (Wash D C). 2024 Nov 1;7:0523. doi: 10.34133/research.0523 (PMC11528066; doi:10.34133/research.0523)
Supplement: Supplementary 1 — Figs. S1 to S9 Tables S1 to S3 [file research.0523.f1.doc]

**Supplementary Information**

**Noninvasive Monitoring of Programmed Death-ligand 2 Expression with PET using ^68^Ga‑labeled Peptide Antagonist in Preclinical and Exploratory Human Studies**

Yajie Zhao^1,#^, Xiaoqin Yin^1,#^, Ming Zhou^1,#^, Wanqian Rao^1^, Xuan Ji^6^, Xiaobo Wang^2*^ XiaoXiong Xiao^3*^ and Shuo Hu^1,4,5*^

1. Department of Nuclear Medicine, Xiangya Hospital, Central South University, Changsha 410008, China.

2. Department of Nuclear Medicine and State Key Laboratory of Holistic Integrative Management of Gastrointestinal Cancers, Xijing Hospital, Fourth Military Medical University, Xi'an 710032, China.

3. Department of Thoracic surgery, Xiangya Hospital, Central South University, Changsha 410008, China.

4. National Clinical Research Center for Geriatric Disorders (Xiangya), Changsha, China.

5. Key Laboratory of Biological Nanotechnology of National Health Commission, Xiangya Hospital, Central South University, Changsha, China.

6. Department of Periodontology, Suzhou Stomatological Hospital, 1366 Suzhan Road, Suzhou, Jiangsu 215026, China.

**^*^Correspondence and reprint request**:

Shuo Hu, M.D., Ph.D., 87 Xiangya Rd., Changsha 410008, China. E-mail: hushuo2018@163.com, Phone/Fax: +86(731)89753869.

XiaoXiong Xiao., Ph.D., 87 Xiangya Rd., Changsha 410008, China. E-mail:xiaoxx1988@csu.edu.cn, Phone/Fax: +86(731)89753003.

Xiaobo Wang, Ph.D., 127 Changle West Road, Xi'an 710032, China. E-mail: xbwang1109@hotmail.com, Phone/Fax: +86(29)-84771048.

**^#^First authors**:Yajie Zhao, Xiaoqin Yin and Ming Zhou contributed equally to this work.

**SUPPLEMENTARY MATERIALS & METHODS**

**General**

All chemicals and solvents were commercially sourced and utilized without further purification. The Sep-Pak QMA and C18 cartridges were procured from Waters (Milford, USA). Radiological analysis was conducted using an Agilent 1260 Infinity II HPLC system (Palo Alto, USA) equipped with a Bioscan flow-count radioactivity detector and a ZORBAX SB-C18 column (5 μm, 4.6 × 250 mm). Radioactivity was measured using a WIZARD 2480 γ-counter (PerkinElmer, USA). For small animal imaging, a nano scan PET/CT system from Mediso (Hungary) was employed. Human imaging and subsequent image analysis were performed using the GE Discovery PET/CT 690 Elite scanner and GE AW 4.6 workstation (Waukesha, USA).

**Radiosynthesis of [^68^Ga]Ga-HN11-1 and [^68^Ga]Ga-HN11-2**

The HN11-1 (5 - 10 nM) and HN11-2 (5 - 10 nM) solutions were transferred into EP tubes with 4.0 mL 0.25 M NaOAc. Subsequently, 1 mL of ^68^GaCl_3_ (30 - 50 mCi) in HCl (0.05 M) was added and the pH of the reaction system was adjusted to 4.0 by adding 0.25 M NaOAc. The reaction mixture was then incubated for 10 minutes at 100 °C. Finally, the final product was obtained through purification using a Sep-Pak C18 cartridge.

**Cell culture, cell transfection and xenograft models**

A549 cell lines were provided by the China Center for Type Culture Collection (CCTCC) and cultured in RPMI-1640 medium supplemented with 10 % fetal bovine serum and 100 U/mL penicillin-streptomycin. The lentivirus system was implemented to introduce PD-L2 (PDCD1LG2) over-expressed cDNA into the A549 cells. The viral vectors were produced in 293T cells and co-transfected with the psAX2, pMD2G and PD-L2 plasmids. A549 cells transduced with the vectors were selectively grown in 1.6 μg/mL puromycin. Transfection efficiency was identified by WB and real-time quantitative PCR. All the cells were cultured in an incubator at 37° C with 5 % CO2 atmosphere. A549 and A549-PD-L2 cells (5 × 10^6^, 200 μL)were subcutaneously implanted into the right shoulder of BALB/C nude mice (male, 4-6 weeks), respectively. When the tumors reached to 5 - 8 mm in diameter, the mice were subjected to in vivo and ex vivo studies. All the mice were purchased from the Laboratory Animal Center of Central South University.

**Cell uptake and binding assays**

All tumor cells (1 × 10^6^ per well) were seeded in two 24 - well plates, incubated for 24 h and then added with 7.40 KBq (0.1 μCi) of [^68^Ga]Ga-HN11-1 or [^68^Ga]Ga-HN11-2 per well. After 15, 30, 60 and 90 min of uptake, the cells were separated from the medium, washed with 500 μL of cold PBS for three times and dissolved with 1 M NaOH. At different time points, these cells were collected and counted by using a γ-counter (WIZARD 2480, Pekin Elmer). For *in vitro* blocking experiments, cells were co-incubated with 10 μg of cold HN11-1 or HN11-2 precursor. After incubation, the cells were washed, collected and measured with a γ-counter, either. The IC_50_ values of [^68^Ga]Ga-HN11-1 or [^68^Ga]Ga-HN11-2 were determined using GraphPad Prism 8.0.2 software, respectively.

**Measurement of Partition Coefficients**

The partition coefficients were assessed using the method described previously[1]. [^68^Ga]Ga-HN11-1 and [^68^Ga]Ga-HN11-2 was respectively introduced into a mixture comprising 3 mL of 1-octanol and 3 mL of phosphate-buffered saline (PBS) with a pH of 7.4. Following vortexing for 10 minutes and subsequent centrifugation at 10,000 rpm for 10 minutes, samples were taken from both layers and counted using a γ-counter. The log *P* was determined using the formula: log *P* = log([M]oct/[M]aq). All measurements were conducted using 5 independent samples and are presented as mean ± standard deviation (SD).

**Stability**

The in vitro stability of [^68^Ga]Ga-HN11-1 and [^68^Ga]Ga-HN11-2 (3.74 MBq) in saline and serum was tested by radio-HPLC analysis of its radiochemical purity after incubation at room temperature for 0.5 , 1 , 2 and 4 h.

**Radio-HPLC Analysis**

The overall radiochemical yield, radiochemical purity, and specific activity were determined using analytical HPLC. The analysis was conducted using an Agilent 1260 Infinity II HPLC system (Palo Alto, USA) equipped with a flow-counter radioactivity detector from BioScan (USA). For the HPLC analysis, a ZORBAX SB-C18 column (5 μm, 4.6 × 250 mm) was employed. The mobile phase consisted of water with 0.1% trifluoroacetic acid (TFA) (A) and acetonitrile with 0.1 % TFA (B). The gradient elution program was as follows: 5 % B from 0 to 2 minutes; linear increase to 90 % B over 15 minutes, with a flow rate of 2 mL/min.

**Radiation Dosimetry Estimation**
The radiation dosimetry assessment of [^68^Ga]Ga-HN11-1 in adult males was conducted by analyzing biodistribution data obtained from BALB/C mice. The uptake percentages (%ID/g) in various tissues and organs were determined and plotted against time to calculate the area under the curves (AUCs) using GraphPad Prism 8.0.2 software. Subsequently, these results were converted to standard human distribution. The absorbed doses for individual organs and the effective dose were estimated utilizing OLINDA/EXM 1.0 software from Hermes Medical Solutions AB, following the Medical Internal Radiation Dose (MIRD) method[2,3].

**Immunohistochemistry for PD-L2 expression**

The formaldehyde-prefixed tumor samples were processed and stained using rabbit primary anti-PD-L2 antibody (Cell signaling technology, CAT # 82723S) as following the standard protocol. After incubation overnight at 4 ℃ and washing with TBST three times, biotinylated goat anti-rabbit IgG (Abcam, ab64256) was added to incubate for 30 min at room temperature. The sections were then exposed to enzyme labeled streptavidin and diaminobenzidine tetrahydrochloride. Finally, the sections were counterstained with hematoxylin and histology images were captured by Zeiss AX10 microscope (Jena, Germany).

**Western blotting for PD-L2 expression**

All cell lysates were separated by 8 % SDS-PAGE and then transferred to 0.45 μm PVDF membranes. The membranes were blocked with 5 % non-fat milk in TBST (0.1 % Tween-20) at room temperature (RT) for 2 h and subsequently incubated with 1:1,000 dilution of anti-PD-L2 antibody (Cell signaling technology, CAT # 82723S) or 1:5,000 dilution of GAPDH (Affinity, CAT # AF0911) antibody at 4℃ overnight. The next day, the membranes were incubated with an HRP-conjugated secondary antibody at RT for 1 h. After washing with TBST three times, the protein bands were visualized by Bio-Rad ChemiDoc MP (Bio-Rad, USA) using an enhanced chemiluminescence reagent (Millipore).

**SUPPLEMENTARY TABLES**

**Supplementary Table 1.** Biodistribution of [^68^Ga]Ga-HN11-1 in A549 tumor xenograft models. The data are shown as mean ± SD (n = 3)

|  | **10 min** | **30 min** | **60 min** | **60 min HN11-1 Block** | **90 min** |
| --- | --- | --- | --- | --- | --- |
| **Kidney** | 55.64 ± 6.60 | 66.15 ± 0.73 | 74.56 ± 3.78 | 76.77 ± 7.10 | 87.01 ± 3.40 |
| **Liver** | 2.90 ± 0.47 | 1.44 ± 0.31 | 0.91 ± 0.14 | 1.20 ± 0.32 | 0.28 ± 0.17 |
| **Blood** | 2.83 ± 0.21 | 1.34 ± 0.16 | 0.34 ± 0.01 | 0.33 ± 0.02 | 0.16 ± 0.03 |
| **Spleen** | 1.91 ± 0.14 | 0.86 ± 0.16 | 0.51 ± 0.17 | 0.96 ± 0.17 | 0.21 ± 0.04 |
| **Heart** | 1.41 ± 0.10 | 0.85 ± 0.10 | 0.18 ± 0.02 | 0.18 ± 0.02 | 0.13 ± 0.01 |
| **Lung** | 1.56 ± 0.06 | 0.96 ± 0.09 | 0.74 ± 0.04 | 0.92 ± 0.04 | 0.40 ± 0.11 |
| **Stomach** | 1.27 ± 0.14 | 0.48 ± 0.04 | 0.19 ± 0.03 | 0.26 ± 0.05 | 0.20 ± 0.02 |
| **Small intestine** | 0.86 ± 0.09 | 0.62 ± 0.04 | 0.21 ± 0.02 | 0.34 ± 0.04 | 0.16 ± 0.05 |
| **Large intestine** | 1.02 ± 0.06 | 0.48 ± 0.09 | 0.21 ± 0.05 | 0.23 ± 0.05 | 0.10 ± 0.01 |
| **Bone** | 0.68 ± 0.06 | 0.60 ± 0.03 | 0.41 ± 0.08 | 0.43 ± 0.07 | 0.31 ± 0.06 |
| **Muscle** | 0.89 ± 0.02 | 0.69 ± 0.07 | 0.56 ± 0.09 | 0.56 ± 0.04 | 0.15 ± 0.03 |
| **Brain** | 0.17 ± 0.03 | 0.12 ± 0.03 | 0.07 ± 0.01 | 0.07 ± 0.01 | 0.03 ± 0.01 |
| **Tumor** | 1.61 ± 0.28 | 3.52 ± 0.35 | 2.20 ± 0.30 | 0.63 ± 0.18 | 0.39 ± 0.00 |
| **Tumor/Heart** | 1.15 ± 0.19 | 4.13 ± 0.21 | 12.72 ± 2.22 | 3.68 ± 1.43 | 3.00 ± 0.15 |
| **Tumor/Liver** | 0.56 ± 0.07 | 2.61 ± 0.82 | 2.53 ± 0.75 | 0.57 ± 0.27 | 2.13 ± 1.27 |
| **Tumor/Lung** | 1.04 ± 0.22 | 3.69 ± 0.24 | 3.01± 0.53 | 0.69 ± 0.23 | 1.05 ± 0.24 |
| **Tumor/Muscle** | 1.82 ± 0.32 | 5.11 ± 0.25 | 4.11 ± 1.25 | 1.11 ± 0.27 | 2.66 ± 0.43 |
| **Tumor/Blood** | 0.58 ± 0.15 | 2.66 ± 0.47 | 6.41 ± 0.98 | 1.94 ± 0.67 | 2.58 ± 0.52 |
| **Tumor/Brain** | 10.34 ± 3.45 | 30.88 ± 8.94 | 31.88 ± 7.97 | 9.41 ± 2.19 | 12.33 ± 2.45 |

**Supplementary Table 2.** A summary of the human equivalent doses for [^68^Ga]Ga-HN11-1. The data are shown as mean ± SD (n = 3).

|  | **Organ Doses (mSv/MBq)** | |
| --- | --- | --- |
| **Target Organ** | **Mean** | **SD** |
| Adrenals | 1.44×10^-2^ | 3.08×10^-3^ |
| Brain | 1.23×10^-3^ | 3.13×10^-4^ |
| Breasts | 5.29×10^-3^ | 2.36×10^-3^ |
| Esophagus | 5.65×10^-3^ | 1.22×10^-3^ |
| Eyes | 4.70×10^-3^ | 1.01×10^-3^ |
| Gallbladder Wall | 7.20×10^-3^ | 1.56×10^-3^ |
| Left colon | 6.39×10^-3^ | 1.37×10^-3^ |
| Small Intestine | 6.17×10^-3^ | 1.32×10^-3^ |
| Stomach Wall | 6.08×10^-3^ | 1.31×10^-3^ |
| Right colon | 6.28×10^-3^ | 1.34×10^-3^ |
| Rectum | 5.81×10^-3^ | 1.24×10^-3^ |
| Heart Wall | 5.95×10^-3^ | 1.28×10^-3^ |
| Kidneys | 9.83×10^-2^ | 2.10×10^-2^ |
| Liver | 1.22×10^-2^ | 2.76×10^-3^ |
| Lungs | 7.34×10^-3^ | 1.64×10^-3^ |
| Ovary | 6.57×10^-3^ | 1.64×10^-3^ |
| Pancreas | 5.91×10^-3^ | 5.47×10^-4^ |
| Prostate | 5.83×10^-3^ | 1.24×10^-3^ |
| Salivary Glands | 5.10×10^-3^ | 1.09×10^-3^ |
| Red Marrow | 4.59×10^-3^ | 9.82×10^-4^ |
| Osteogenic Cells | 4.22×10^-3^ | 9.04×10^-4^ |
| Spleen | 7.17×10^-3^ | 2.38×10^-3^ |
| Testes | 5.12×10^-3^ | 1.09×10^-3^ |
| Thymus | 5.43×10^-3^ | 1.17×10^-3^ |
| Thyroid | 5.33×10^-3^ | 1.14×10^-3^ |
| Urinary Bladder Wall | 5.70×10^-3^ | 1.22×10^-3^ |
| Uterus | 7.38×10^-3^ | 2.46×10^-3^ |
| Total Body | 5.99×10^-3^ | 1.29×10^-3^ |
| Effective Dose | 5.79×10^-3^ | 1.25×10^-3^ |

**Supplementary Table 3** Patient Characteristics.

| **Patient NO.** | **Gender** | **Age**  **(y)** | **tumor type^*^** | **Primary tumor site** | **PD-L2**  **expression** | **FDG**  **SUV_max_** | **HN11-1**  **SUV_max_** |
| --- | --- | --- | --- | --- | --- | --- | --- |
| 1 | Female | 68 | NSCLC | Lung | 80 % | 15.5 | 4.1 |
| 2 | Male | 41 | NSCLC | Lung | 15 % | 25.9 | 2.1 |
| 3 | Male | 57 | rHNSCC | buccal | / | 13.7 | 4.0 |
| 4 | Male | 50 | rHNSCC | buccal | / | 4.0 | 3.0 |
| 5 | Male | 60 | mHNSCC | tongue | / | 4.0 | 2.7 |
| 6 | Male | 51 | mHNSCC | laryngeal | / | 3.7 | 3.6 |

* NSCLC, Non-small cell lung cancer;

rHNSCC, recurrent head and neck squamous cell carcinoma;

mHNSCC, metastatic head and neck squamous cell carcinoma.

**SUPPLEMENTARY FIGURES**

**
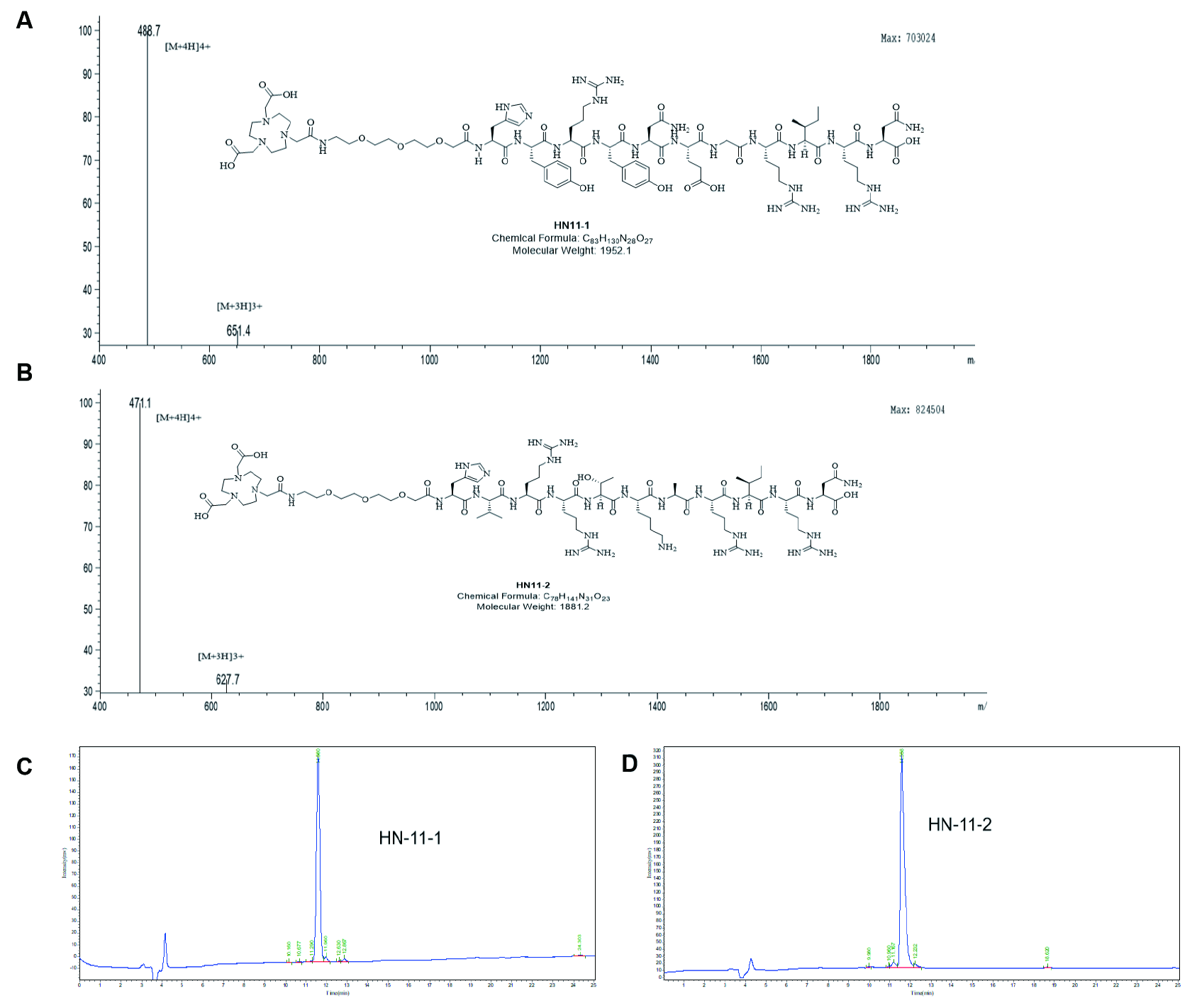
**

**Supplementary Figure 1.** MS spectrum of HN11-1 (A) and HN11-2 (B), HPLC of HN11-1 (C) and HN11-2 (D).


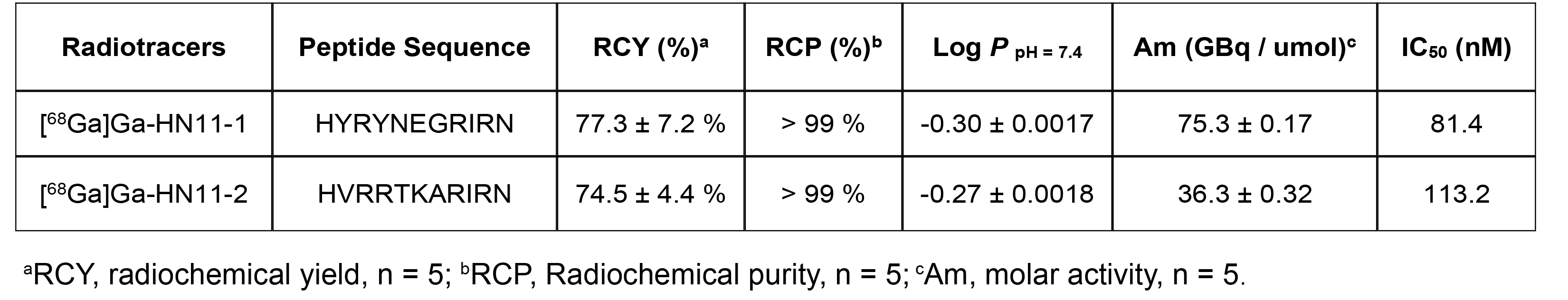
**Supplementary Figure 2.** Radiochemical characterization and binding affinity of [^68^Ga]Ga-HN11-1 and [^68^Ga]Ga-HN11-2.

**
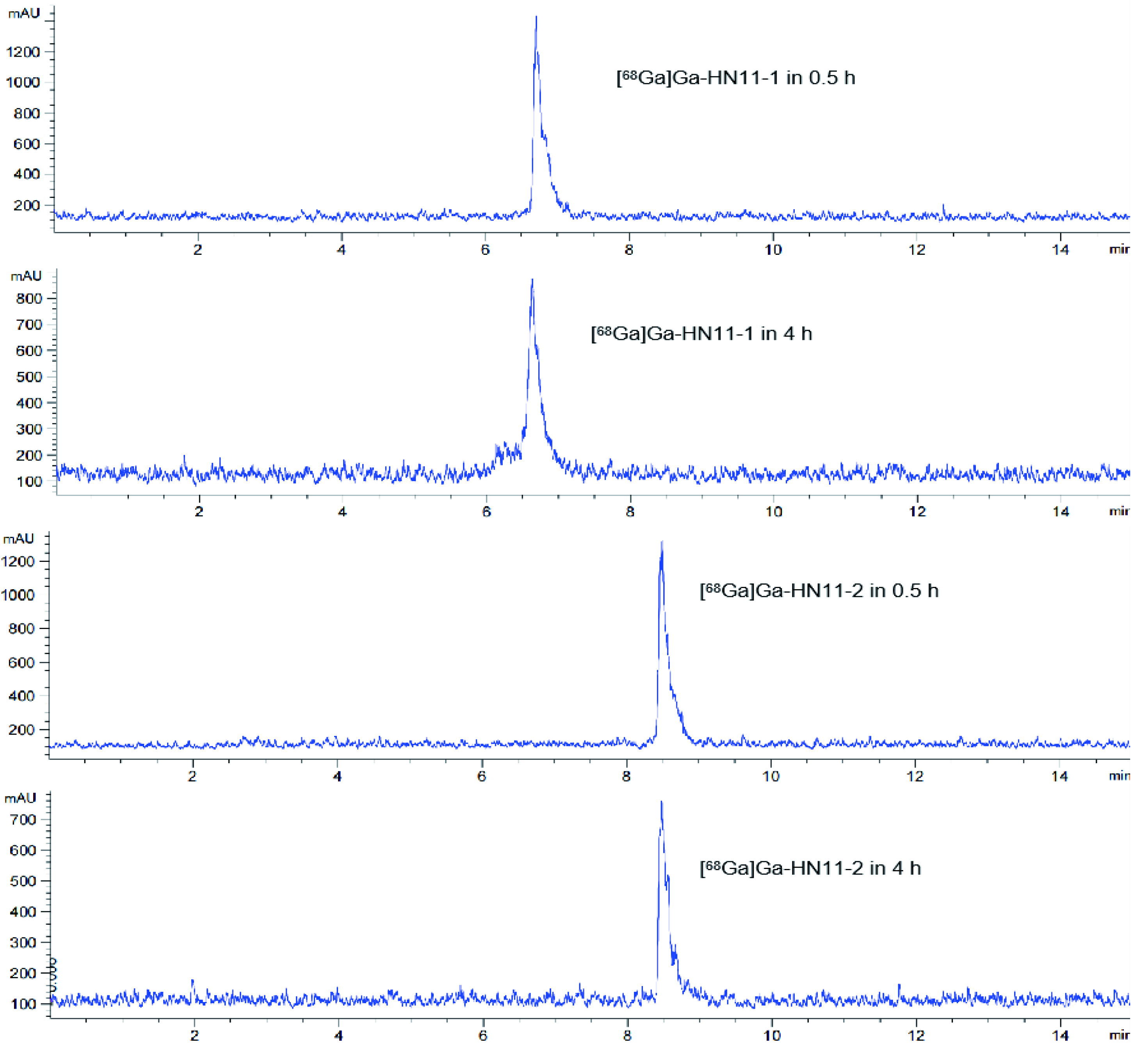
Supplementary Figure 3.** Analytical radio-HPLC chromatogram of [^68^Ga]Ga-HN11-1 and [^68^Ga]Ga-HN11-2 in 0.5 h and 4 h.


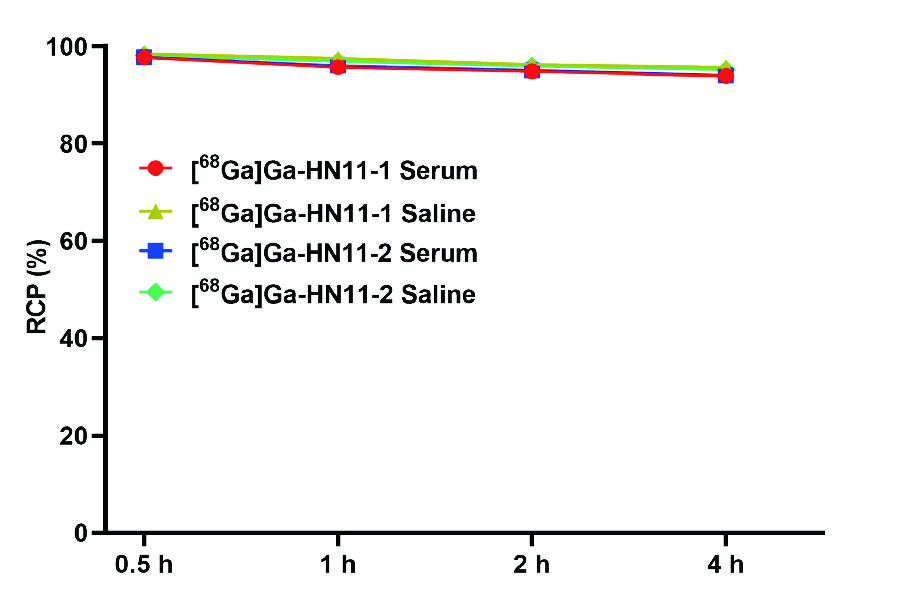
**Supplementary Figure 4.** *In* *vitro* stability of [^68^Ga]Ga-HN11-1 and [^68^Ga]Ga-HN11-2 in saline and serum.

**
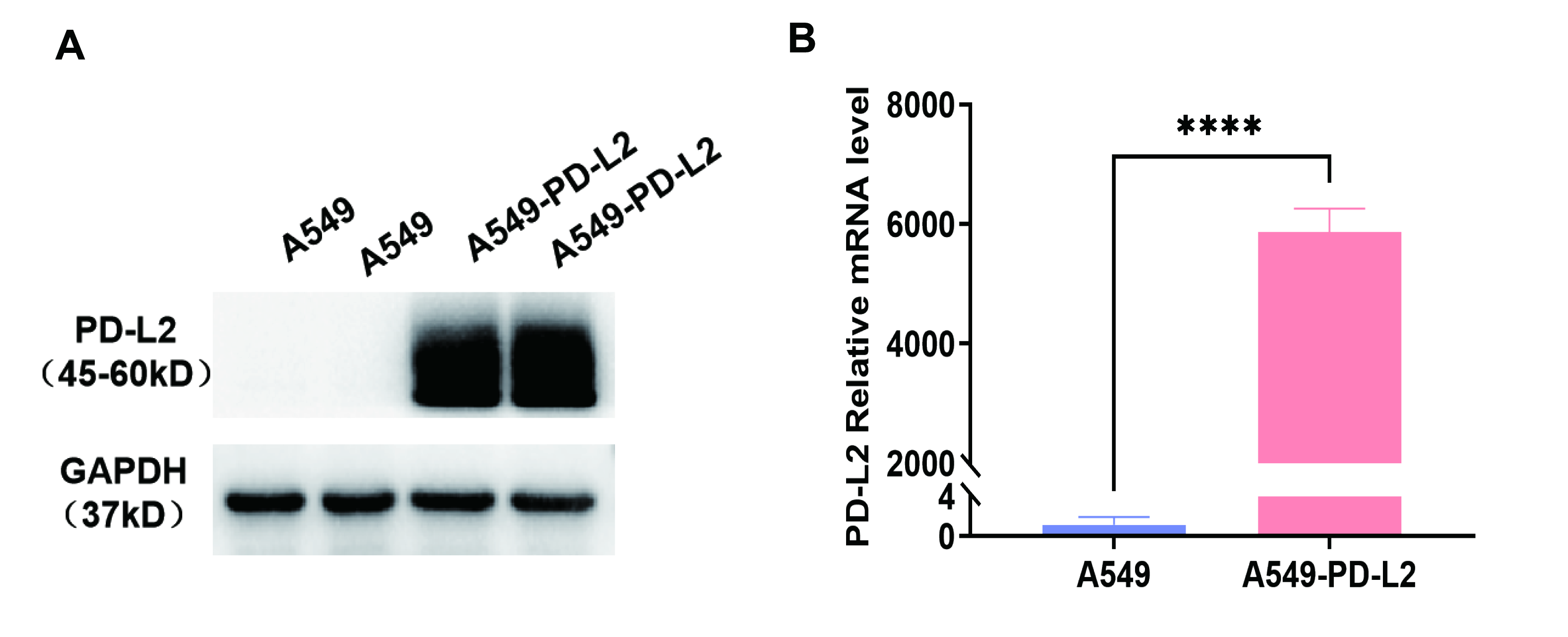
Supplementary Figure 5.** (A) WB verified the transfection of PD-L2 in A549 cells. (B) q-PCR verified the transfection of PD-L2 in A549 cells.


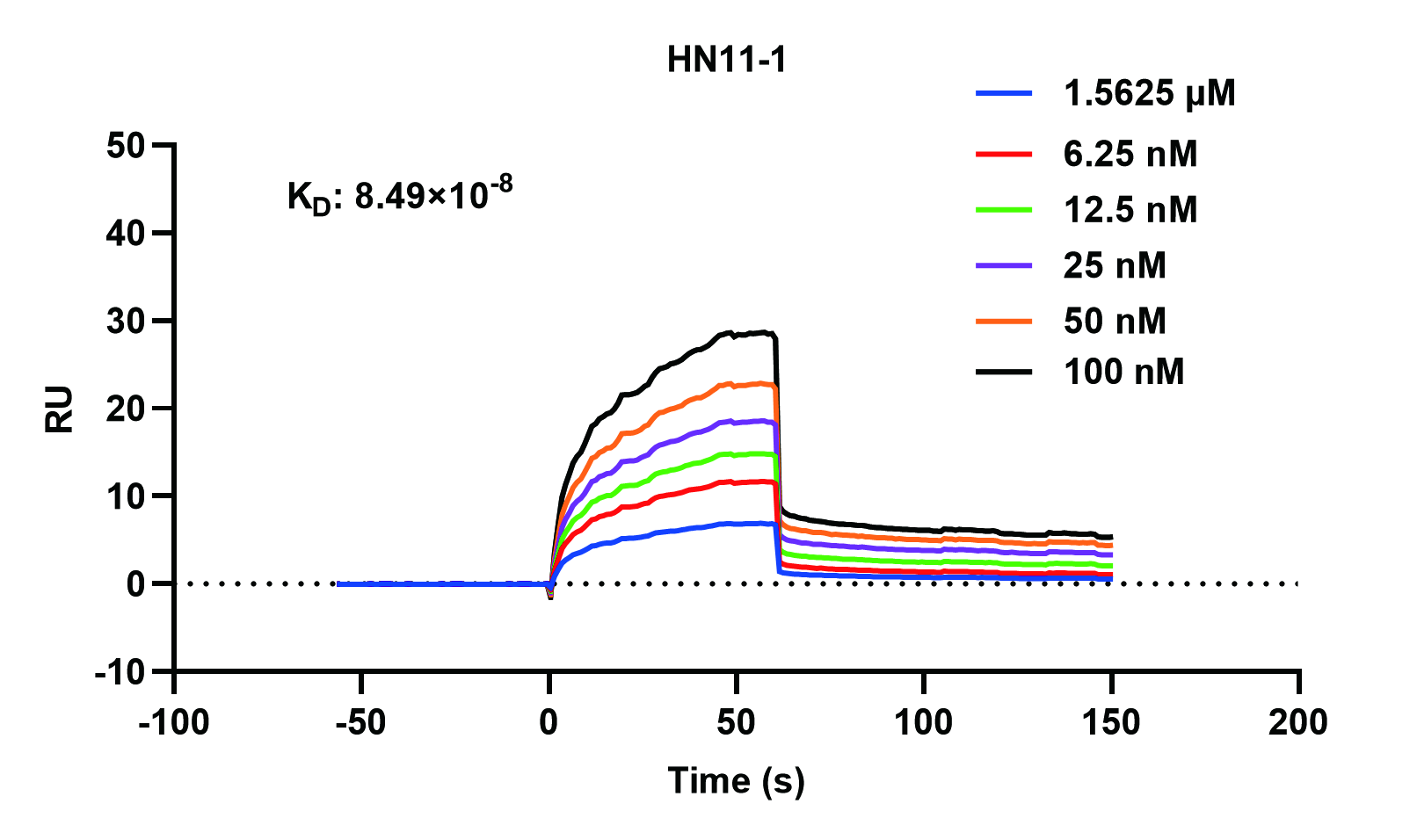


**Supplementary Figure 6.** Surface Plasmon Resonance (SPR) of HN11-1.


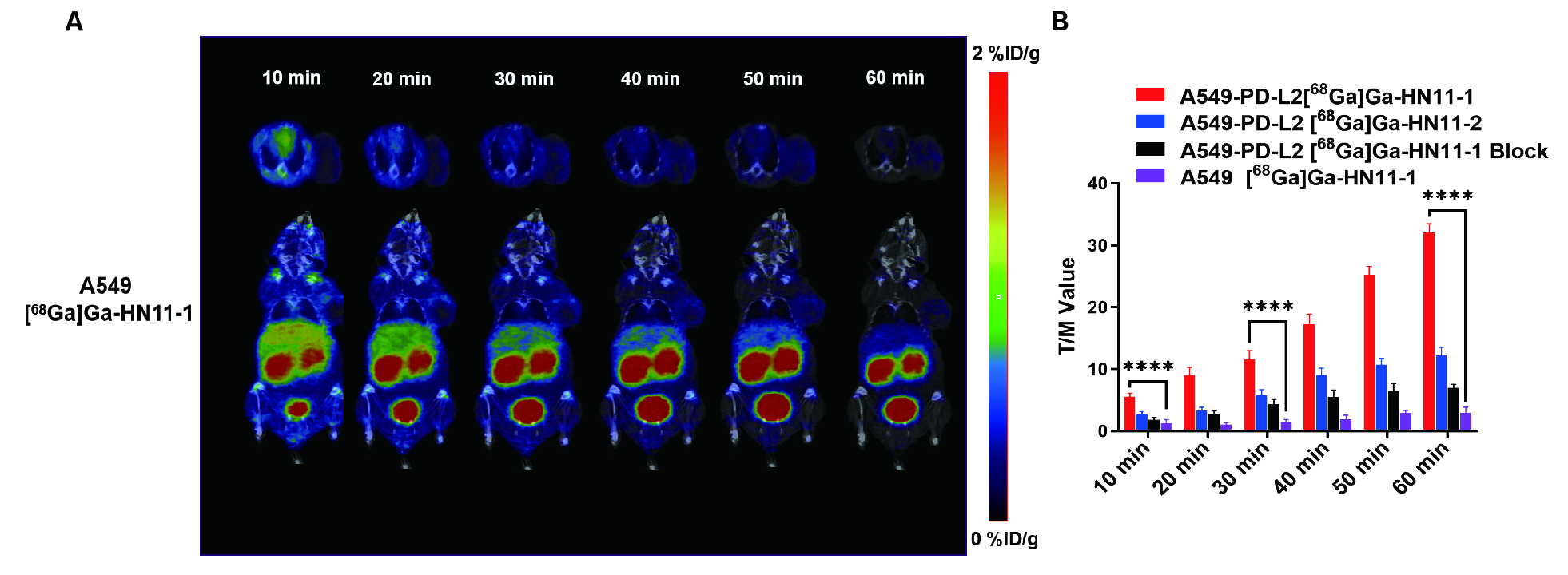
**Supplementary Figure 7.** (A) Dynamic PET imaging at different time points in A549 xenograft models after injection of [^68^Ga]Ga-HN11-1 (n = 3). (B) The T/M ratios of [^68^Ga]Ga-HN11-1, [^68^Ga]Ga-HN11-2, [^68^Ga]Ga-HN11-1 + block in A549-PD-L2 xenograft models, and [^68^Ga]Ga-HN11-1 in A549 xenograft models at 10, 20, 30, 40, 50 and 60 min (n = 3).


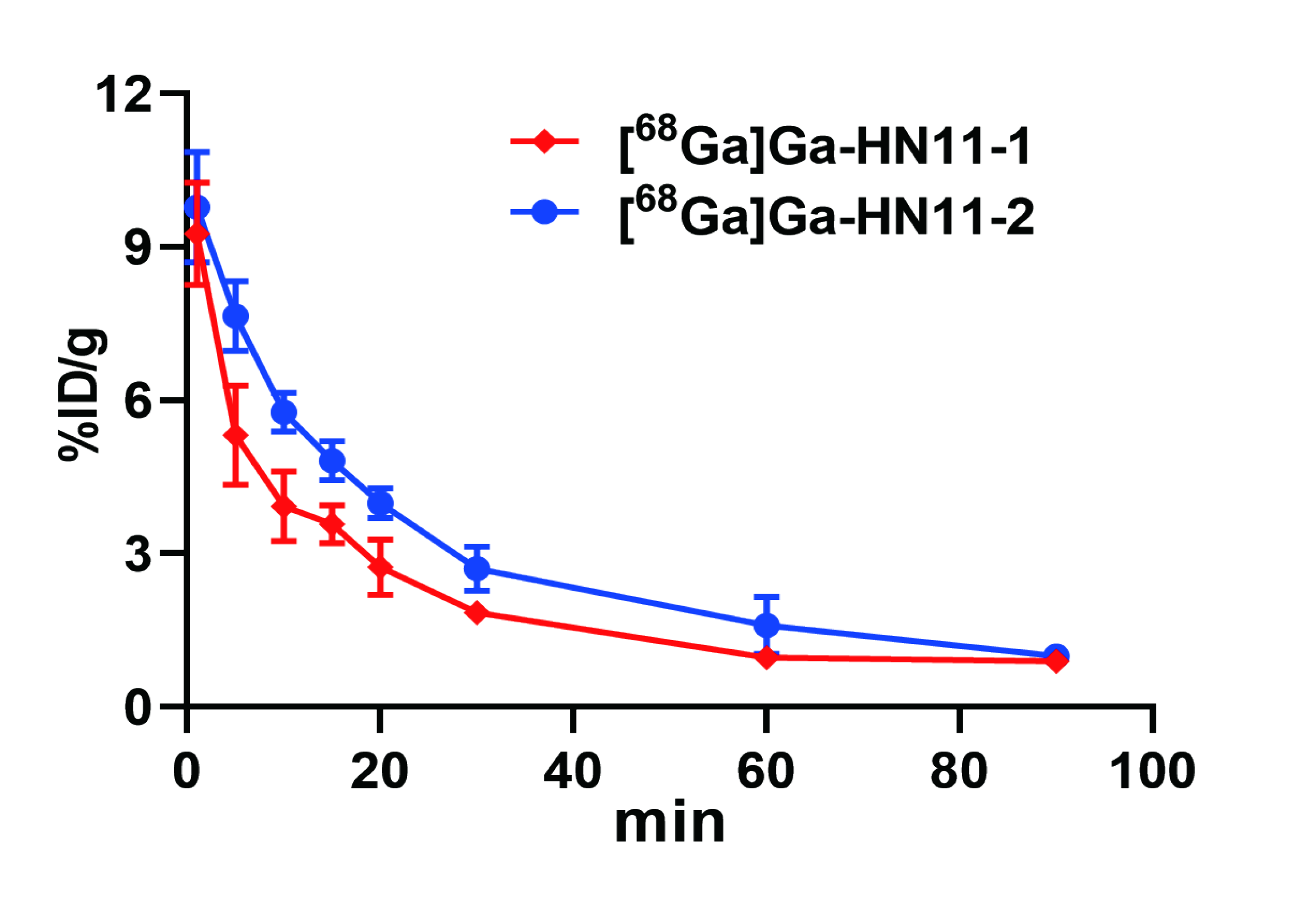


**Supplementary Figure 8.** The pharmacokinetics of [^68^Ga]Ga-HN11-1 and [^68^Ga]Ga-HN11-2 in blood at 20, 40, 60 ,80 and 100 min, respectively.


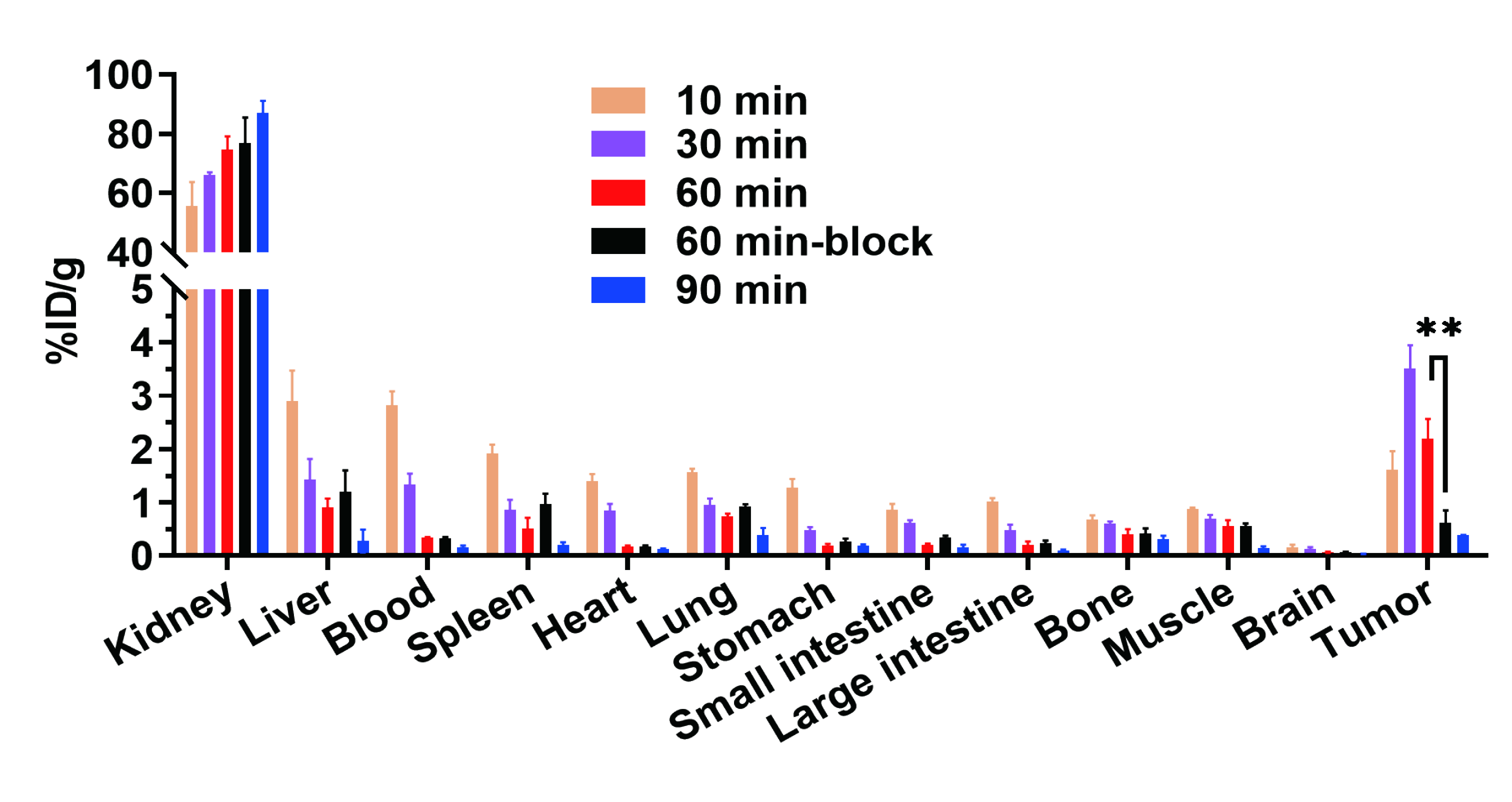


**Supplementary Figure 9.** Biodistribution of [^68^Ga]Ga-HN11-1 in major organs at 10, 30, 60 and 90 min, and [^68^Ga]Ga-HN11-1 + block in major organs at 60 min (n = 3).

**References**

[1] Z. Zhang, X. Wang, J. Ye, H. Liu, J. Fang, M. Zhang, Y. Li, J. Huang, D. Zhang, J. Wang, X. Zhang, *Mol Pharm* 2021, *18*, 3509.

[2] Taylor DM. Generic models for radionuclide dosimetry: ^11^C-, ^18^F- or ^75^Se-labelled amino acids. *Appl Radiat Isot.* 2000;52:911-22.

[3] S. Wang, X. Zhou, X. Xu, J. Ding, S. Liu, X. Hou, N. Li, H. Zhu, Z. Yang, *Eur J Nucl Med Mol Imaging* 2021, *48*, 4259.
